# Recent Development of Functional Bio-Based Epoxy Resins

**DOI:** 10.3390/molecules29184428

**Published:** 2024-09-18

**Authors:** Yuan Zhang, Xuemei Liu, Mengting Wan, Yanjie Zhu, Kan Zhang

**Affiliations:** Institute of Polymer Materials, School of Materials Science & Engineering, Jiangsu University, Zhenjiang 212013, China

**Keywords:** bio-based epoxy resin, flame retardant, recyclable, shape memory, antibacterial

## Abstract

The development of epoxy resins is mainly dependent on non-renewable petroleum resources, commonly diglycidyl ether bisphenol A (DGEBA)-type epoxy monomers. Most raw materials of these thermoset resins are toxic to the health of human beings. To alleviate concerns about the environment and health, the design and synthesis of bio-based epoxy resins using biomass as raw materials have been widely studied in recent decades to replace petroleum-based epoxy resins. With the improvement in the requirements for the performance of bio-based epoxy resins, the design of bio-based epoxy resins with unique functions has attracted a lot of attention, and bio-based epoxy resins with flame-retardant, recyclable/degradable/reprocessable, antibacterial, and other functional bio-based epoxy resins have been developed to expand the applications of epoxy resins and improve their competitiveness. This review summarizes the research progress of functional bio-based epoxy resins in recent years. First, bio-based epoxy resins were classified according to their unique function, and synthesis strategies of functional bio-based epoxy resins were discussed, then the relationship between structure and performance was revealed to guide the synthesis of functional bio-based epoxy resins and stimulate the development of more types of functional bio-based epoxy resins. Finally, the challenges and opportunities in the development of functional bio-based epoxy resins are presented.

## 1. Introduction

Epoxy resin is a three-dimensional polymer network with insoluble and insoluble properties generated after the cross-linking reaction of epoxy monomer or oligomer with a curing agent [1,2,3]. Epoxy resin has the advantage of excellent thermal and mechanical properties, chemical resistance, and dimensional stability, and it can be applied in aerospace, coatings, high-performance composites, electronic encapsulants, and other fields [4,5,6,7]. At present, more than 90% of the world’s epoxy resin is composed of bisphenol A diglycidyl ether (DGEBA), formed from bisphenol A (BPA) and epichlorohydrin (ECH) [1]. Among them, commercial ECH is derived from bio-based glycerol. However, BPA is derived from non-renewable petroleum resources, and excessive use will cause an energy crisis [8]. In addition, BPA is harmful to our health [9]. Due to concerns about health, energy, and the environment, finding suitable biomass as raw materials to replace petroleum-based BPA has become a research hotspot. At present, vegetable oil, itaconic acid (IA), cardanol, and other biomass have been used for the preparation of bio-based epoxy resins [10,11,12,13,14].

For a long time, the purpose of studying the preparation of bio-based epoxy resin was to find suitable biomass raw materials, so that the performance of the prepared bio-based epoxy resin is comparable to that of petroleum-based epoxy resin, so as to replace petroleum-based epoxy resin. Under the above purpose, some bio-based epoxy resins with similar properties to petroleum-based epoxy resins have been designed [15,16,17,18,19,20,21]. A typical example is the trifunctional epoxy monomer from IA denoted TEIA. TEIA monomer is liquid and has low viscosity (0.92 Pa s, 25 °C) for easy processing. The methylhexahydropthalic anhydride was used as a curing agent to cure TEIA and a petroleum-based epoxy monomer to obtain the corresponding resin. The experimental results showed that TEIA had higher reactivity, and TEIA-based epoxy resin exhibited a higher glass transition temperature (*T*_g_, 135.2 °C vs. 109.5 °C) and a higher flexural strength (157.2 MPa vs. 131.6 MPa) than that of the DGEBA system [18]. More efforts have been made in the research and development of bio-based epoxy resins, as well as the gradual improvement of the requirement for material performance. More and more bio-based epoxy resins with better performance (high *T*_g_, high degradation temperature, high tensile strength, high energy storage modulus) than petroleum-based epoxy resin have been reported [22,23,24,25,26,27,28]. These bio-based epoxy resins mainly use magnolol, resveratrol, vanillin, ferulic acid, etc., as raw materials [22,23,24,25,26,27,28].

**Scheme 1:**
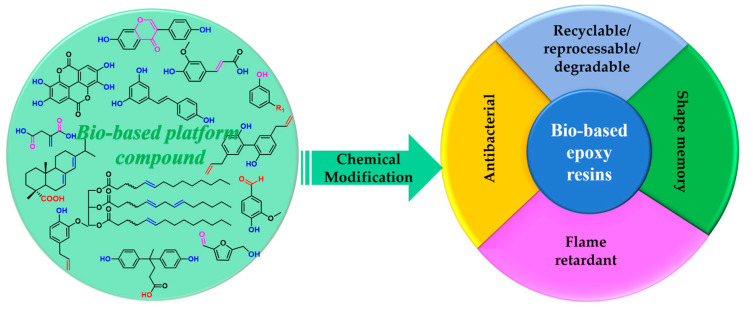
The process from bio-based platform compounds to functional bio-based epoxy resins.

Although bio-based epoxy resins with better thermal and mechanical properties have been designed and prepared, there are some drawbacks in bio-based epoxy resin that make it impossible to produce on a large scale, such as the high cost of biomass feedstock and the difficulty of processing bio-based epoxy monomers [29,30,31,32,33,34,35,36,37]. In order to overcome these drawbacks and improve competitiveness, bio-based epoxy resins should not only meet the requirements of better thermal and mechanical properties, but also should be designed to explore their unique functionality, including flame-retardant performance [29,30,31,32], degradability, recyclability, reprocessability [33,34,35], shape memory function [33,34,35], antibacterial properties [36], etc. [37]. This class of bio-based epoxy resins with unique functions has attracted wide attention, and a lot of studies have been conducted in recent years [29,30,31,32,33,34,35,36,37]. Although there are a large number of reviews on the preparation of bio-based epoxy resins from biomass, most of the reviews focus on their high performance rather than on their functions [2,3,4,5,6,7,10,11,12]. Some reviews only focus on one kind of function, such as flame retardancy, of bio-based epoxy resins [29,30,31,32]. A comprehensive review of the functional bio-based epoxy resins is still lacking. This review aims to summarize the recent development of bio-based epoxy resins with different functions derived from biomass (Scheme 1). On this basis, there is a need to summarize the structural characteristics and synthesis methods of functional bio-based epoxy resins, and reveal the relationship between structure and function to provide methods for the design and synthesis of functional bio-based epoxy resins in the future. Finally, the challenges and opportunities of functional bio-based epoxy resins are discussed.

## 2. Synthesis Methods of Various Functionalized Bio-Based Epoxy Resins

**Scheme 2:**
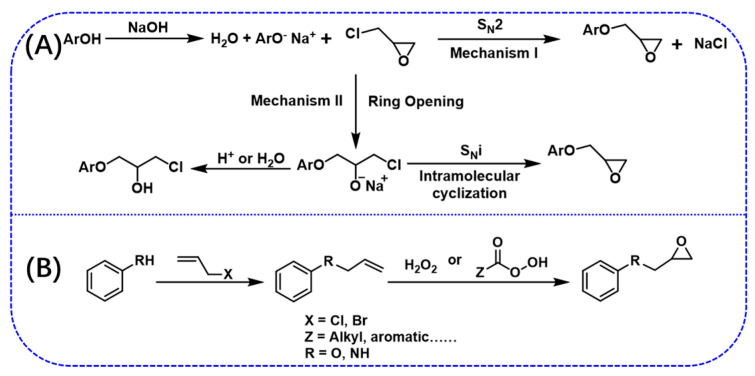
Synthesis of epoxy resins from phenolic compounds via glycidylation reaction (**A**) and double-bond oxidation (**B**).

The synthesis of commonly used epoxy monomers is shown in Scheme 2. One approach to obtaining the bio-based epoxy monomers is the glycidylation reaction between plant phenols and excess ECH using a phase transfer catalyst in alkaline conditions (Scheme 2A). Another method for the preparation of bio-based monomers is the peroxidation of a carbon–carbon double bond in biomass employing the H_2_O_2_ or stronger oxidative reagents such as m-chloroperbenzoic acid (m-CPBA) (Scheme 2B) [29].

The synthesis of functional bio-based epoxy monomers is also achieved by the above two methods. For the synthesis of flame-retardant bio-based epoxy resins, flame-retardant elements such as phosphorus [30], or specific structures that can produce a high char yield at high temperatures, such as rigid and conjugate structures or heterocyclic structures, need to be introduced in the intermediate [31]. For the synthesis of recyclable/degradable/reprocessable bio-based epoxy resins, in the first method, polyphenol intermediates containing dynamic chemical bonds, such as imine bonds, disulfide bonds, etc., are usually synthesized first, and then react with ECH [33,34,35]. In the second approach, the introduction of a bio-based epoxy curing agent with dynamic covalent bonds or the formation of reversible dynamic covalent bonds (such as ester bonds) during the curing process are also valid methods to prepare recyclable bio-based epoxy resins [33,34,35]. In addition, the introduction of dynamic bonds is also a common method to prepare bio-based epoxy resins with shape memory function [33,34,35]. Moreover, the introduction of long alkyl chains in the network structure of epoxy resins can also be used to design shape memory epoxy resins [36]. The synthesis of antibacterial bio-based epoxy resins is usually achieved by introducing groups with antibacterial effects such as a hydroxyl group and Schiff base [36].

## 3. Functional Bio-Based Epoxy Resins

### 3.1. Flame Retardant

Bio-based epoxy resins have the same or better performance than petroleum-based epoxy resins, but they also suffer from the disadvantage of being flammable, which limits their application in areas, such as the electrical and electronic industries where flame retardancy is needed [29,30,31,32]. Therefore, new bio-based epoxy resins with flame retardancy have been widely developed. The bio-based epoxy resin with intrinsic flame retardancy refers to giving itself flame-retardant properties by introducing suitable biomass or a flame-retardant monomer into the epoxy resin network by covalent bonds. The main methods usually include (1) introducing flame-retardant elements such as P [30], and (2) introducing biomass having a high char yield [31]. The bio-based flame-retardant epoxy resins exert their flame-retardant effect through several mechanisms, such as capturing gas-phase free radicals, coverage effect, dilution effect, endothermic effect, and synergy effect. Generally, many bio-based flame-retardant epoxy resins achieve the flame-retardant effect through the joint action of several mechanisms [29,30,31,32]. When burned, a compact and continuous char layer covering the surface of polymeric materials is formed, which has the functions of heat insulation, oxygen isolation, and preventing flammable gas from escaping outward, to achieve the purpose of flame retardancy. In this process, a large number of non-combustible gases (CO_2_, N_2_, NH_3_, etc.) can be produced, so that the concentration of oxygen and other combustible gases produced by the decomposition of epoxy resins are diluted and cannot reach the lower limit of the combustion concentration range in the combustion zone. Furthermore, epoxy resins undergo decomposition or other endothermic reactions under high-temperature conditions, which reduce the temperature of epoxy resins and combustion zone, and effectively inhibit the generation of combustible gas and continuous combustion reaction [29,30,31,32]. For phosphorus-containing flame-retardant bio-based epoxy resins, active free radicals such as PO•, PO_2_•, and HPO_2_• are generated during thermal decomposition and combustion process, which capture free radicals such as H• and HO• in the gas phase to generate table compounds, reducing the concentration of combustible free radicals in the gas phase and hindering the continuous combustion of epoxy resin [30]. At present, flame-retardant bio-based epoxy resins are divided into two categories: phosphorus-containing and phosphorus-free according to the flame-retardant mechanism.

#### 3.1.1. Phosphorus-Containing Flame-Retardant Bio-Based Epoxy Thermosets

**Scheme 3:**
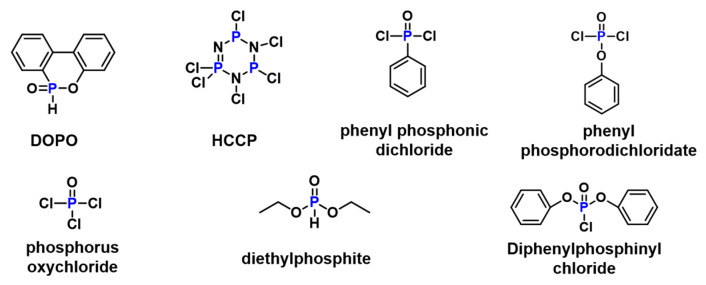
Chemical Structure of commonly used phosphorus-containing flame retardants.

Flame retardants containing phosphorous, for instance, 9,10-dihydro-9-oxo-10-phosphaphenanthrene-10-oxide (DOPO) [38,39,40,41,42,43], hexachlorocyclotriphosphazene (HCCP) [44,45], and various phosphates [46,47,48,49,50,51,52,53,54,55,56,57] (Scheme 3), have gained great popularity in contemporary society due to their low toxicity, high efficiency, multiple flame-retardant mechanisms, and molecular diversity. Some research advances about phosphorus-containing flame-retardant bio-based epoxy thermosets are shown in Table 1 (yellow part), and some typical examples are briefly described. The research progress of phosphorus-containing flame-retardant bio-based epoxy resin is classified and discussed below, according to the different sources of phosphorus.

**Scheme 4:**
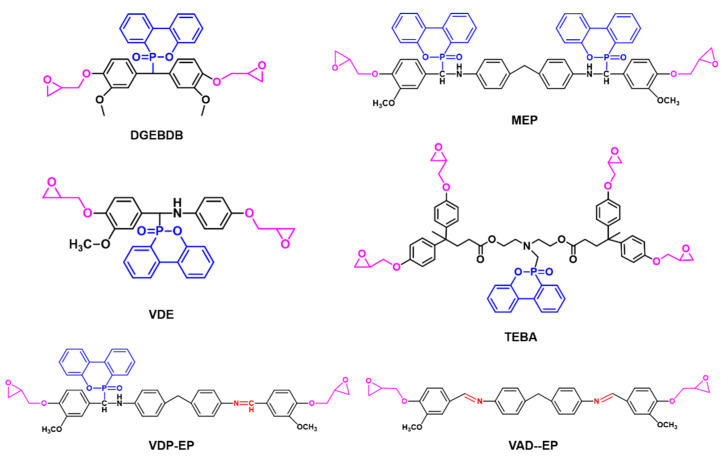
The chemical structure of some bio-based epoxy monomers containing DOPO, and contrast of VAD-EP.

**Figure 1:**
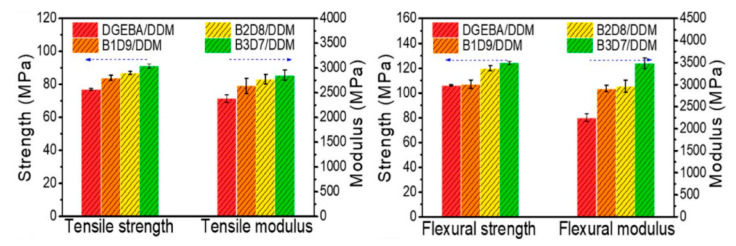
Mechanical properties of all the cured resins with different weight ratios of DGEBDB/DGEBA. Reproduced with permission from ref. [39]. Copyright 2020 Elsevier.

**Figure 2:**
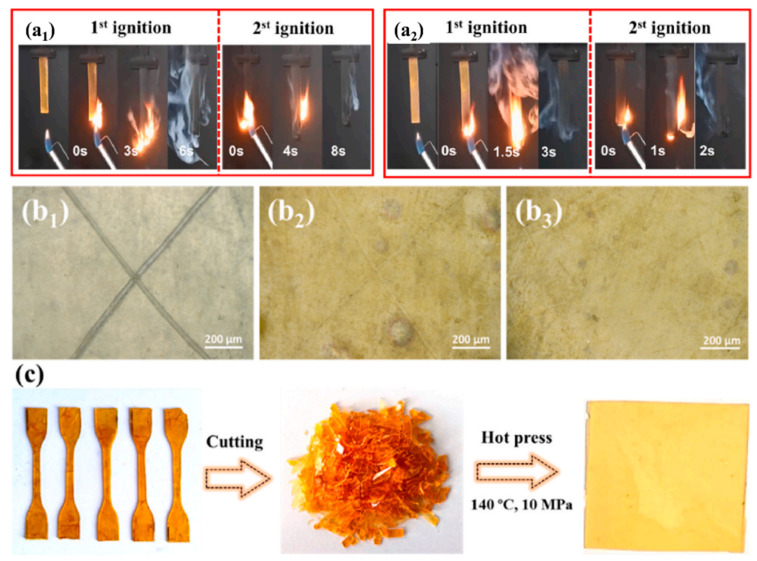
Digital photographs of vertical combustion of cured epoxy VAD-EP(8)-VDP-EP(2)-D230 (**a_1_**) and VAD-EP(7)-VDP-EP(3)-D230 (**a_2_**); Digital photos demonstrating self-healing (**b**, subscript 1, 2 and 3 represent 0 h, 12 h and 24 h, respectively) and reprocessability (**c**) of VAD-EP(7)-VDP-EP(3)-D230. Reproduced with permission from ref. [43]. Copyright 2022 Elsevier.

**Phosphaphenanthrene-based bio-based epoxy resin.** DOPO is one of the most commonly used phosphorus-containing flame-retardant compounds, with high thermal stability and good oxidation resistance. DOPO and its derivatives are often used to synthesize phosphorus-containing epoxy resins due to its structure containing an active P-H bond [38,39,40,41,42,43] (the chemical structure of some bio-based epoxy monomers containing DOPO is shown in Scheme 4). For example, Liu et al. used a simple method to synthesize a phosphorous-containing bio-based epoxy monomer of DGEBDB from vanillin and guaiacol, as shown in Scheme 4 [39]. Then, DGEBDB was mixed with petroleum-based epoxy DGEBA and cured with 4,4-diaminodiphenyl methane (DDM) to obtain cured epoxy resin. The results showed that DGEBDB and DGEBA have good compatibility, and when the weight ratio of DGEBDB and DGEBA reached 2/8, the cured epoxy resin exhibited good flame retardancy and passed the UL-94 V-0 rating, and the combustion results are summarized in Table 1. Moreover, the mechanical properties of epoxy resin after adding DGEBDB are obviously improved (Figure 1). The mechanism of DGEBDB in improving the flame retardancy of cured resin was attributed to the char layers in the condensed phase. If the DOPO-containing epoxy resin is modified with other functional groups, the epoxy resin can possess both flame retardancy and other functions. For example, Ma and co-workers synthesized two novel epoxy monomers of VAD-EP and VDP-EP from vanillin (Scheme 4) [43]. Then, two epoxy monomers were mixed in different proportions and cured with a D230 diamine curing agent to obtain epoxy resin. When the ratio of VAD-EP and VDP-EP is 8/2, the cured epoxy resin has good flame retardancy, which has passed the UL-94 V-0 test (Figure 2a_1_), and the combustion results are summarized in Table 1. The flame retardancy is attributed to the condensed phase and free radical capture. When the weight ratio of VAD-EP and VDP-EP is 7/3, the cured epoxy resin is not only flame-retardant but also has good self-healing and reprocessing ability (Figure 2a_2_,b,c), and 86.1% of the tensile strength of the material can be retained after one reprocessing. And this resin was completely degraded by stirring in 1M HCl/THF(2/8) solution at 60 °C for 1 h. The recyclability of the resin enables it to be applied to carbon-fiber-reinforced composite materials to achieve the non-destructive recovery of carbon fiber.

**Scheme 5:**
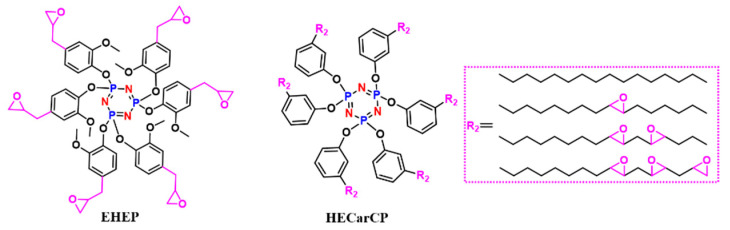
The chemical structure of some bio-based epoxy monomers containing HCCP.

**Cyclotriphosphazene-based bio-based epoxy resin.** It is well known that P and N play a synergistic role in flame retardancy and have an obvious self-extinguishing effect. In addition, flame retardants containing P and N are considered environmentally friendly. Therefore, HCCP with alternating P and N in the ring structure (Scheme 3) has been extensively studied for the synthesis of flame-retardant polymers [44,45]. For example, Lei and co-workers synthesized six functional epoxy monomers of EHEP (Scheme 5) with eugenol and HCCP as raw materials, and a cured EHEP monomer with a D230 diamine hardener. The results showed that the resin of EHEP-D230 had good flame retardancy, passing the UL-94 V-0 rating, because the expanded carbon-layer structure formed during the combustion process played a role in flame retardancy and smoke suppression. In addition, EHEP-D230 has higher tensile strength (64.2 vs. 56.7 MPa) and better heat resistance (*T*_g_ = 119 vs. 85 °C) compared to the commercial epoxy of E51-D230 [44]. Soon afterward, Hu and co-workers also synthesized bio-based epoxy resin with flame-retardant properties using cardanol and HCCP as raw materials [45], and the structure of this cardanol-derived epoxy monomer of HECarCP is shown in Scheme 5, and the combustion results are summarized in Table 1.

**Scheme 6:**
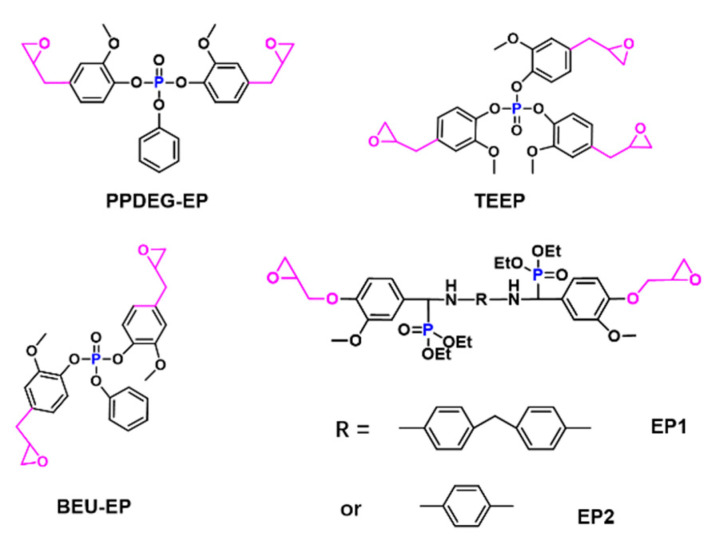
The chemical structure of some phosphonate- and phosphate-based bio-based epoxy monomers.

**Figure 3:**
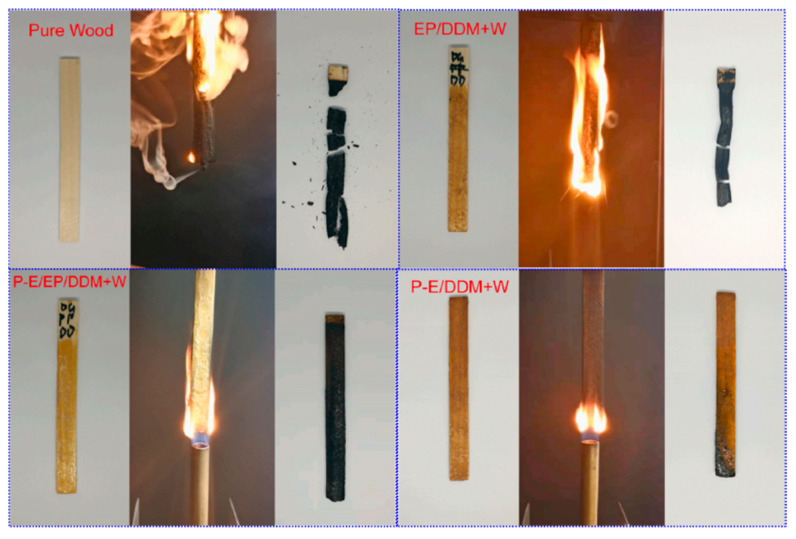
Photographs of pure wood splines and different epoxy-coated splines before, during, and after the UL-94 test. W is short for wood, EP is short for BPA-based epoxy resin. Reproduced with permission from ref. [48]. Copyright 2022 Elsevier.

**Phosphonate- and phosphate-based bio-based epoxy resin.** Phosphonate- and phosphate-based epoxy monomers have been synthesized for the development of epoxy resins with inherent flame-retardant properties (the chemical structure of some phosphonate- and phosphate-based bio-based epoxy monomers is shown in Scheme 6) [46,47,48,49,50,51,52,53,54,55,56,57]. For example, Guo et al. synthesized a novel epoxy monomer named PPDEG-EP (P-E) from eugenol and phenylphosphonic dichloride, as shown in Scheme 6. And the results showed that the cured PPDEG-EP by DDM, PPDEG-EP/DDM, has a lower reaction activation energy (*E*_a_) than commercial epoxy resin. This bio-based epoxy resin containing phosphorus could be used as a coating for wood (Figure 3), which not only retained the physical and chemical properties of commercial epoxy thermoset but also exerted flame-retardant properties [48]. In another work, Zhu et al. reported the synthesis of two epoxy monomers (EP1 and EP2, Scheme 6) from vanillin, two diamines, and diethyl phosphate. The results showed that the reactivities of these two epoxy monomers are similar to that of commercial epoxy monomers, and both the cured epoxy resins of EP1 and EP2 have flame-retardant properties (UL-94 V-0), and EP2 has higher *T*_g_ (214 vs. 166 °C), tensile strength (80.3 vs. 76.4 MPa) and tensile modulus (2709 vs. 1893 MPa) compared to those of the commercial epoxy resin [54].

#### 3.1.2. Phosphorus-Free Flame-Retardant Bio-Based Epoxy Thermosets

Although the main components of phosphorus-free bio-based epoxy resin are C, H, O, and N, which do not contain flame-retardant elements (P, Si, etc.), the flame-retardant performance can still be endowed to the phosphorus-free bio-based epoxy resin through appropriate selection or design of biomass structure [29,31]. Some research advances about phosphorus-free flame-retardant bio-based epoxy thermosets are shown in Table 1 (blue part), and some typical examples are briefly described. The key to the flame-retardant performance of bio-based epoxy resin is to embed a special structure that produces a high char yield at high temperatures in the polymer networks. When the flame-retardant bio-based epoxy resin is heated or burned, the release of non-flammable gases (CO_2_, NH_3_, etc.) will dilute the oxygen and take away some of the heat to inhibit combustion. The dense and stable char layer formed during the combustion process acts as a barrier to heat transfer and protects the interior of the resin [29]. Biomass containing rigid and conjugated structures usually possesses a higher char yield at high temperatures [31]. According to the chemical structure, flame-retardant bio-based epoxy resins are divided into rigid/conjugated structure and heterocyclic structure, and the recent research progress is summarized and discussed, respectively.

**Scheme 7:**
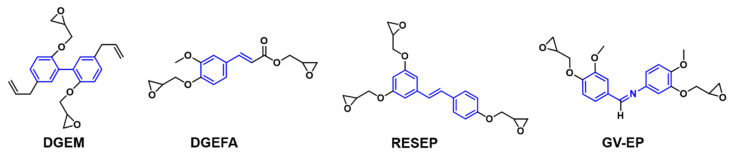
The chemical structure of some representative epoxy monomers with a rigid and conjugated structure.

**Rigid and conjugated structure.** Some of the bio-based epoxy monomers with a rigid and conjugated structure are directly synthesized from biomass raw materials including magnolol, resveratrol, ferulic acid, etc. [58,59,60,61,62,63,75,76,77,78,79]. The chemical structure of some representative epoxy monomers with a rigid and conjugated structure is shown in Scheme 7. Magnolol, containing symmetrical and reactive allyl groups and phenolic hydroxyl groups, is extracted from the bark of magnolia officinalis. Because of its rigid and conjugated structure, it has been used many times to synthesize flame-retardant bio-based epoxy resins [58,59,75,76]. For example, Weng et al. synthesized magnolol-derived epoxy monomer of DGEM (Scheme 7) by reacting magnolol with excessive epichlorohydrin. The cured DGEM/DDS was obtained after curing with DDS. The DGEM/DDS resin exhibited a high char yield of 42.8% and showed excellent flame-retardant performance (UL-94 V-0 rating) due to the dense and stable carbon layer produced during combustion. In addition, the resin also has a higher *T*_g_ (279 vs. 231 °C) and higher mechanical modulus and strength compared to the commercial epoxy DGEBA/DDS [58]. Moreover, the same authors also fabricated the tetra-functional epoxy monomer of MTEP derived from magnolol by successive reactions of magnolol with epichlorohydrin and epoxidation of allyl. The cured MTEP/DDS with higher cross-linking than DGEM/DDS also possessed outstanding intrinsic flame retardancy, and the combustion results are summarized in Table 1 [59]. Resveratrol with conjugated stilbene structure is extracted from grape skin, peanuts, giant knotweed, etc. There are three phenolic hydroxyl groups in the structure of resveratrol, which react with epichlorohydrin to synthesize epoxy monomer [60,78,79]. Zheng and co-workers reported a resveratrol-based epoxy resin (RESEP, Scheme 7) cured by DDM. The *T*_g_ and the char yield (800 °C) of RESEP/DDM were higher than those of DGEBA/DDM (335 °C vs. 168 °C, 38.6% vs. 16.9%). Typically, RESEP/DDM displayed a UL-94 V-0 rating [60]. Biomass that has similar conjugated structure of phenylidene to resveratrol and has been used for the synthesis of flame-retardant bio-based epoxy resins also includes ferulic acid [61], 4-coumaric acid [62], curcumin [63], and so on. Moreover, they have a lower initial degradation temperature (Table 1) due to the presence of easily broken bonds in the structure at high temperatures [61,62,63].

The direct use of biomass to prepare bio-based epoxy resin can no longer meet the material requirements, so the design of bio-based epoxy resin with good comprehensive properties through molecular modification has been widely reported. Inspired by the direct use of biomass to prepare flame-retardant bio-based epoxy, a rigid and conjugated structure plays an important role in flame retardants, so the molecular design with conjugated structure is carried out [64,65,66,80,81,82,83]. Schiff bases, which refer to compounds with the structure of R_2_C=NR′(R′H) formed by the addition reaction of aldehyde or ketone with primary amine, are commonly used to introduce into polymers for the preparation of flame-retardant bio-based epoxy resins. At high temperature, the internal -CH=N- bonds of the bio-based epoxy-containing imine bonds will be rearranged and cyclized, resulting in nitrogen-containing six-membered heterocyclic compounds. These heterocyclic compounds can make polymer chains self-crosslink, thus enhancing the char-forming ability of materials and improving the flame-retardant properties of materials [64,65,66,80]. For example, Weng et al. synthesized a bifunctional epoxy monomer of GV-EP (Scheme 7) containing a Schiff base using vanillin and guaiacol as raw materials. Then, GV-EP was cured with DDM to obtain the GV-EP/DDM. This epoxy resin network with a dynamic Schiff base structure (GV-EP/DDM) exhibited a higher *T*_g_ (220 vs. 187 °C) char yield (44.9% vs. 12.8% at N_2_ at 800 °C) and storage modulus (3602 MPa vs. 2604 MPa) than commercial bisphenol A-based epoxy resin, and possessed inherent flame-retardant properties (UL-94 V-0 rating). Furthermore, GV-EP/DDM also has satisfactory degradability, reprocessing, and antibacterial properties due to the introduction of the Schiff base structure [64].

**Scheme 8:**
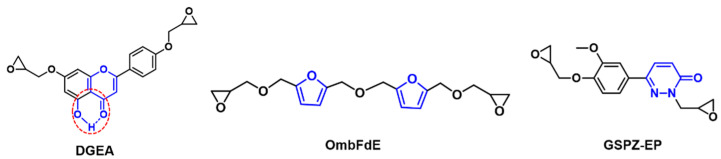
The chemical structure of some representative epoxy monomers with heterocyclic structure.

**Figure 4:**
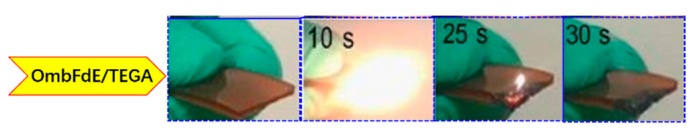
Burning test of OmbFdE/TEGA furan-based epoxy resin. Reproduced with permission from ref. [87]. Copyright 2019 The Royal Society of Chemistry.

**Heterocyclic structures.** The most representative biomass containing a heterocyclic structure is flavonoids including daidzein, apigenin, genistein, and luteolin. Flavonoids, which contain two or more phenolic hydroxyl groups, have been directly reacted with epichlorohydrin to prepare bio-based epoxy monomers [67,68,69,84,85,86]. For example, Hu and co-workers synthesized an apigenin-based epoxy monomer (DGEA) using an efficient one-step process (Scheme 8), and cured it with DDM. The results indicated that the cured DGEA/DDM system possessed the *T*_g_ of up to 232 °C (156 °C for DGEBA/DDM), and showed higher storage modulus and tensile strength than those of cured DGEBA/DDM, which was ascribed to the high cross-link density of cured DGEA caused by the dimerization of the benzopyrone ring and the active role of intramolecular hydrogen bonds. The cured DGEA/DDM system demonstrated excellent flame-retardant properties, showing a residual char of 45.1% at 700 °C, limiting oxygen index (LOI) of 37.0%, and flammability rating of V-0 in the UL-94 test [85]. Other flavonoid-derived epoxy resins also exhibited outstanding flame-retardant properties, and the related data about their performance are summarized in Table 1. Heterocyclic structure embedding in bio-based epoxy resin through molecular modification can also be used to prepare flame-retardant bio-based epoxy resin [70,71,72,73,74,87,88,89,90,91]. Furan and its derivatives are often used to design flame-retardant bio-based epoxy resins containing a heterocyclic structure [71,87,88,89,90,91]. For example, Guo et al. designed a bis-furan diepoxide called OmbFdE (Scheme 8), which was eco-friendly. The flame retardancy of the cured OmbFdE/2,2′-(ethane-1,2-diylbis(oxy)) bis(ethan-1-amine) (TEGA) was investigated, and the results exhibited that the OmbFdE/TEGA possessed outstanding flame retardancy (Figure 4), which was attributed to a comparable lower heat release [87]. Additionally, Weng et al. synthesized an epoxy monomer of GSPZ-EP containing a heterocycle structure (Scheme 8) using biomass guaiacol and malanhydride as raw materials. After the curing process with DDM, the obtained GSPZ-EP/DDM showed higher *T*_g_ (187 vs. 173 °C), storage module (3327 ± 38 MPa vs. 1952 ± 20 MPa, at 30 °C), and char yield (42.3% vs. 17.8%, in N_2_ at 700 °C) compared with commercial DGEBA/DDM. Moreover, GSPZ-EP/DDM has excellent inherent flame-retardant properties, and its flame-retardant rating is close to UL-94 V-0 [72].

### 3.2. Recyclable/Reprocessable/Degradable Bio-Based Epoxy Thermosets

Epoxy resins have a lot of advantages, such as excellent mechanical properties, chemical resistance, weather resistance, good electrical insulation, and dimensional stability, so it has been widely used in composite materials [1,2,3]. However, they suffer from unsustainability because of their non-reprocessability, being difficult to recycle, and being excessively dependent on petroleum chemicals [33]. The recent development of bio-based epoxy resins containing dynamic bonds provides an alternative solution for the sustainable development of conventional petroleum-based epoxy resins, because they are not only independent of petroleum chemicals but can be reprocessed and recycled by dynamic bonds under suitable conditions [33,34,35,36]. Various dynamic bonds have been employed, such as ester bonds [92,93,94,95,96,97,98,99,100,101,102,103,104,105,106,107,108,109] and similar structures [110,111,112], Schiff bases [113,114,115,116,117,118,119,120,121,122,123,124], acetal structures [125,126,127], Diels–Alder addition structures [128,129,130,131,132,133,134], and disulfide bonds [135,136,137,138,139,140,141]. The transesterification of the ester bonds occurs easily through the breaking of C-O bonds at high temperature. In addition, hydrolysis of the ester bond can be catalyzed in an acidic or alkaline environment. Therefore, the ester bond is one of the dynamic covalent bonds usually used to prepare recyclable thermosetting resins [92,93,94,95,96,97,98,99,100,101,102,103,104,105,106,107,108,109]. The Schiff base is also one of the dynamic covalent bonds commonly used to prepare recyclable thermosetting resins. Schiff bases promote the recyclability/degradability of epoxy resin mainly through three dynamic reactions, including imine–amine exchange (transamination), imine–imine exchange (imine metathesis), and imine condensation/hydrolysis [113,114,115,116,117,118,119,120,121,122,123,124]. The acetal structure is unstable under acidic conditions and decomposes into the original aldehydes (ketones) and alcohols, thus achieving the degradability of the epoxy resin [125,126,127]. D-A reaction occurs at a lower temperature (50–80 °C) to generate a cyclohexene adduct, and the reverse reaction of the D-A reaction, retro-D-A reaction, occurs at higher temperatures (110–140 °C) to form the starting material adiene and conjugated diene. The introduction of the D-A structure into the epoxy resin makes the epoxy resin recyclable by changing the temperature [128,129,130,131,132,133,134]. The mechanical force can cause the disulfide bond to break and produce the thiol radical, which can be quickly exchanged with other disulfide bonds to achieve the reprocessability of the cross-linked network [135,136,137,138,139,140,141]. In this part, we summarize the recent progress on the design and synthesis of bio-based epoxy resins containing various dynamic bonds.

#### 3.2.1. Ester Bonds and Similar Structures

Ester bonds are reversible covalent bonds that are unstable under high-temperature, acidic, or alkaline conditions [92,93,94,95,96,97,98,99,100,101,102,103,104,105,106,107,108,109]. Transesterification occurs through the breaking of the C-O bond at high temperature [99,100,101,102,103,104,105,106,107,108,109], and the hydrolysis of ester bonds can be catalyzed in an acidic or alkaline environment [92,93,94,95,96,97,98]. Therefore, the degradation and recycling of epoxy resin can be achieved under certain conditions by introducing an ester bond structure in the molecule [92,93,94,95,96,97,98,99,100,101,102,103,104,105,106,107,108,109].

**Figure 5:**
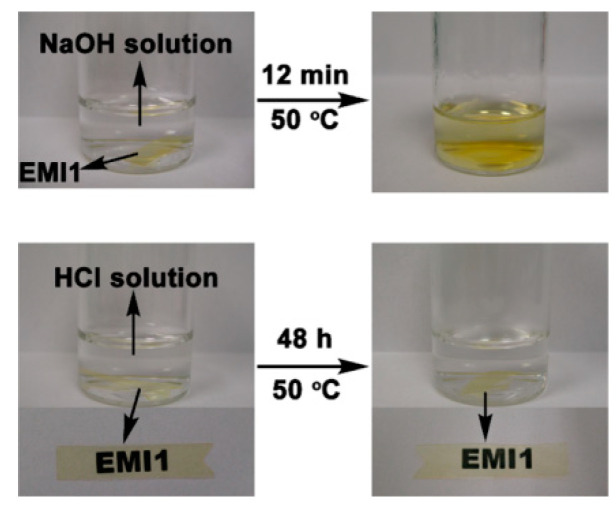
Appearance of EMI1 before and after immersing in 1 M NaOH aqueous solution (**top**) or 1 M HCl aqueous solution (**bottom**). Reproduced with permission from ref. [96]. Copyright 2016 American Chemical Society.

**Scheme 9:**
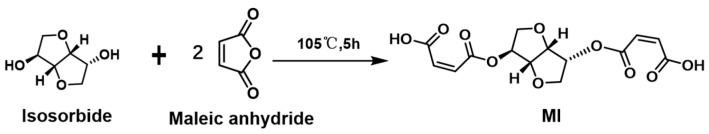
Synthetic route of a dicarboxylic acid oligomer (MI).

**Scheme 10:**
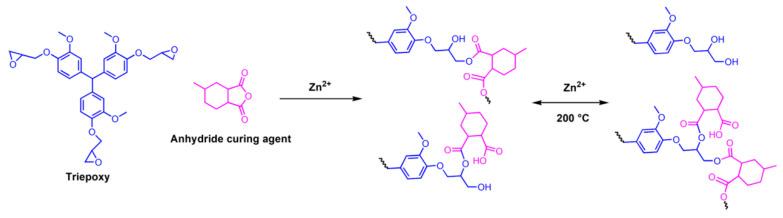
Curing reaction of TEP and anhydride and the illustration of dynamic transesterification reactions in the cross-linked network structure.

**Figure 6:**
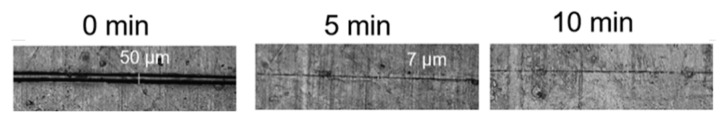
Thermal repairing of cured TEP by clipping the samples between two metal plates and heating in a convection oven without press (stoichiometric ratio between epoxy group and anhydride group is 1/1). Reproduced with permission from ref. [100]. Copyright 2018 American Chemical Society.

For example, Ma et al. prepared a dicarboxylic acid oligomer named MI from isosorbate and maleic anhydride by the route shown in Scheme 9, which was then used as a curing agent for epoxidized sucrose soybean meal (ESS) to obtain the thermosetting resin called EMI 1. At 50 °C, EMI 1 was stable in 1 m HCl aqueous solution; in contrast, it was degraded and completely dissolved at 12 min in 1 M NaOH aqueous solution, as shown in Figure 5. The carboxylic acid resulting from the hydrolysis of the ester bond under alkaline conditions was neutralized by the alkali to form the carboxylate, so the epoxy resin containing the ester bond has better degradability under the catalysis of the base. In the cured epoxy resin, the hydroxyl group produced by the ring-opening reaction can undergo a dynamic transesterification reaction with the ester bond at high temperature (>150 °C), which makes the material repairable and reprocessable [96]. In addition, excessive zinc salts are usually used to catalyze transesterification in industry [99,100,101,102,103,104,105,106]. For example, Liu et al. synthesized a bio-based triepoxy called TEP from vanillin and guaiacol, and the cured TEP by an anhydride exhibited high performance and rapid self-healing capability through dynamic transesterification. The width of the crack from cured TEP can be effectively repaired within 10 min without pressure (Figure 6). It is worth noting that a zinc catalyst was chosen to catalyze the curing reaction and also used as the catalyst for transesterification in the cured TEP (Scheme 10) [100].

**Scheme 11:**
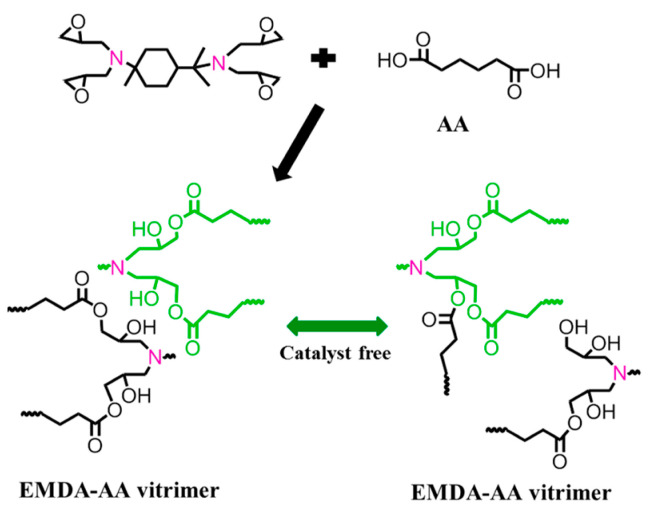
The curing reaction of the EMDA-AA vitrimer. An illustration of dynamic transesterification reactions in the cross-linked network structure.

If the cured epoxy material structure contains tertiary amines, the transesterification reaction can be performed without a metal catalyst, and the recycle reaction can also occur under mild conditions [107,108,109]. For example, Chen and co-workers prepared a bio-based epoxy resin of epoxidized menthane diamine–adipic acid (EMDA-AA, Scheme 11) containing ester bonds from menthane diamine and adipic acid biomass. The EMDA-AA resin can be rearranged in the network structure by the dynamic transesterification reaction without additional catalyst (Scheme 11). And the EMDA-AA matrix in the composite material can be degraded into polyols by amination reactions under mild conditions, and realize the recycling of fibers [107].

The borate ester bond (B-O-C), as a dynamic reversible chemical bond, has similar properties to the ester bond. On the one hand, the boron ester, performing an associative transesterification reaction in the permanent cross-linked network, enables the rearrangement of the network that contributed to the reprocessing of cured resins in the solid state. On the other hand, borate ester bonds are sensitive to reactive oxygen species, and in acidic media with pH much lower than its pKa, borate ester bonds are more easily hydrolyzed into boric acids and diols, which is conducive to the recycling of resins containing borate ester bonds [110,111,112]. Therefore, the boron ester bond provides a path for designing recyclable/reprocessable/degradable bio-based epoxy resin.

**Scheme 12:**
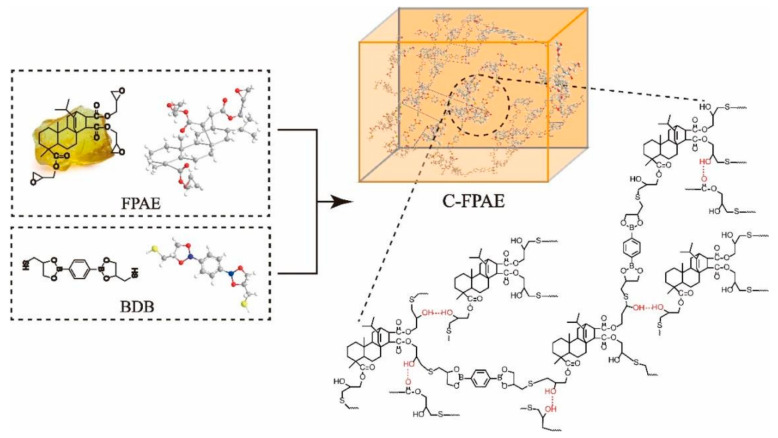
Synthesis of a C-FPAE cross-linking network.

**Figure 7:**
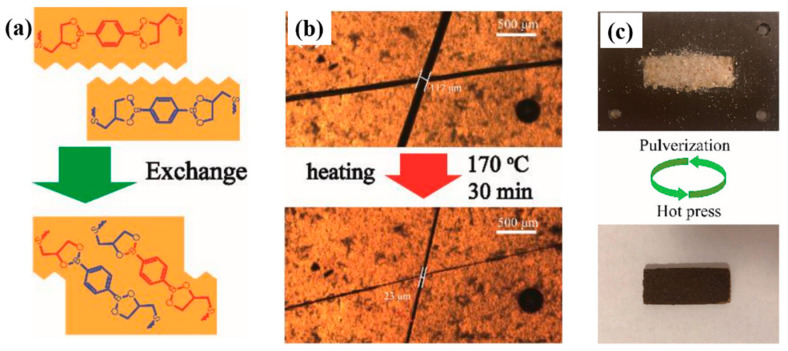
(**a**) Schematic representation of the transesterification reaction of boronic ester linkages, optical microscope images of self-healing performance (**b**) and thermal recycling performance of 20% C-FPEA (**c**). Reproduced with permission from ref. [110]. Copyright 2021 MDPI.

**Scheme 13:**
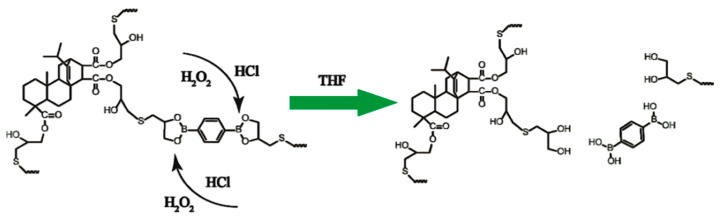
Schematic representation of C-FPAE degradation.

For example, Zeng et al. fabricated a bio-based epoxy vitrimer called C-FPAE, containing dynamic reversible boronic ester bonds using rosin derivative (FPAE) as an epoxy monomer and 2,2′-(1,4-phenylene)-bis(4-mercaptan-1,3,2-dioxaborolane) (BDB) as a hardener (Scheme 12) [110]. The C-FPAE has superior thermostability and thermomechanical properties due to the presence of the structure of the rosin derivative, enhanced covalent cross-linker, and the hydrogen bond in the networks (Scheme 12). Moreover, C-FPAE possesses recyclable and self-healing properties through the transesterification reaction of boron ester bonds in the network structure (Figure 7a). For example, a 20% C-FPEA (x% C-FPAE refers to cured FPAE with x mol% of BDB) sheet sample with a thickness of 0.7 mm was cut into a width of 117 μm. The cut samples were healed at 170 °C in an oven for 30 min. As shown in Figure 7b, the cut 20% of C-FPEA samples achieved 80% recovery after healing treatment, showing good self-healing ability. And the 20% C-FPAE solid was ground into powder and hot-pressed at 200 °C for 60 min to obtain a uniform sample, exhibiting good reprocessing properties (Figure 7c). In addition, C-FPEA samples can also be degraded in active oxygen and acidic conditions. At 30 °C, 20% C-FPAE powder samples were soaked in THF/H_2_O_2_/HCI mixture with different pH and stirred for 48 h. As pH decreases from 6.5 to 0.0, the degradation weight percentage increases from 10% to 90%, and the degradation mechanism is shown in Scheme 13. It follows that C-FPAE samples are more easily degraded in strong acid solution [110].

**Scheme 14:**
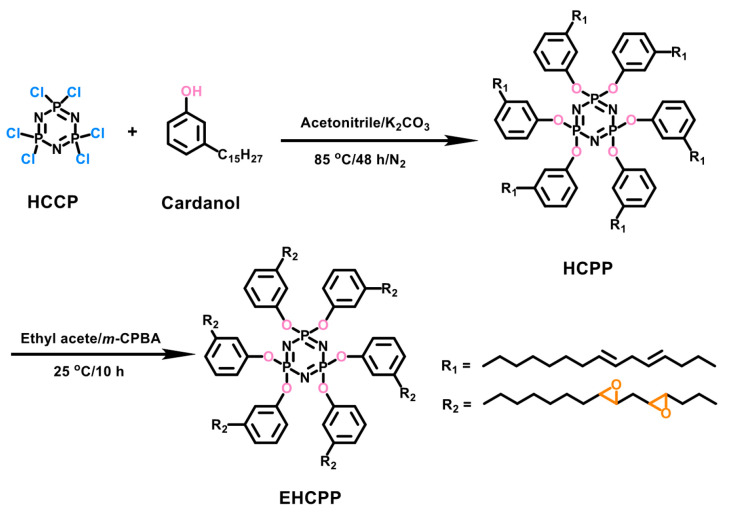
Synthetic route of EHCPP.

**Figure 8:**
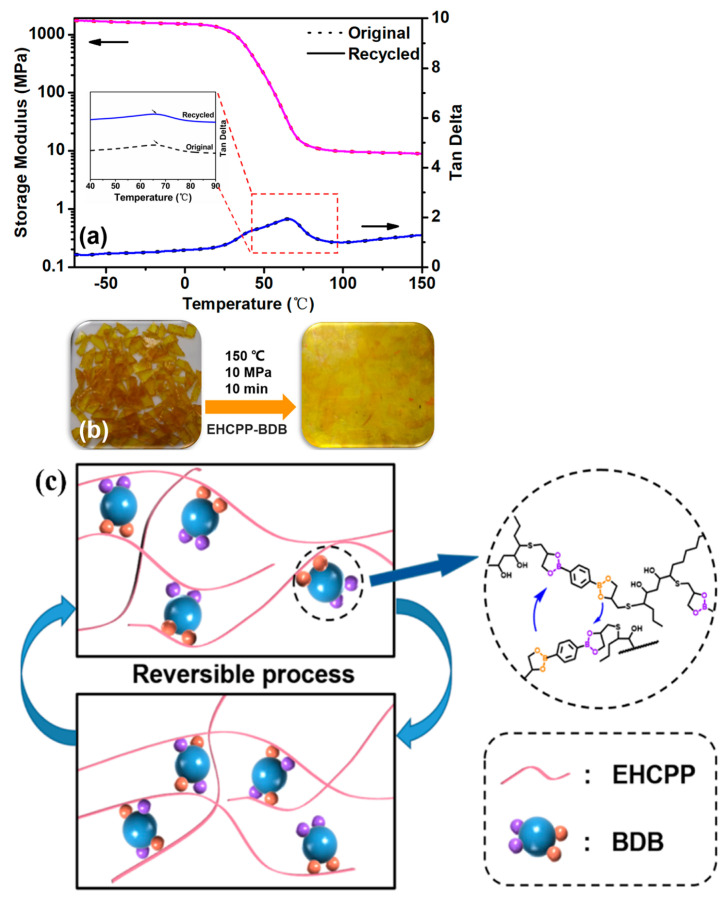
(**a**) DMA of the original and recycled EHCPP-BDB polymer networks (**b**) photographs of original and reshaped EHCPP polymer networks; (**c**) Schematic illustration of the reversible mechanism of boron ester in BDB (SH monomer) of the cardanol-based epoxy polymer networks. Reproduced with permission from ref. [112]. Copyright 2021 American Chemical Society.

Similarly, Zhang et al. also utilized BDB as a curing agent to cure the cardanol-derived epoxy monomer of EHCPP (the synthetic route and structure of EHCPP are shown in Scheme 14) to prepare EHCPP-BDB epoxy polymer networks containing borate ester bonds [112]. EHCPP-BDB polymer was cut into small pieces and then placed into the mold and remolded to new samples by hot-pressing at 150 °C under a pressure of 10 MPa for 10 min, and the EHCPP-BDB was remolded to form a continuous and uniform sample (Figure 8b), and dynamic mechanical analysis (DMA) studies showed that the original and remolded samples had a similar *T*_g_ and storage moduli (at 25 °C), exhibiting excellent reprocessability (Figure 8a). The reversible mechanism of boron ester in the cardanol-based epoxy polymer network is shown in Figure 8c. In addition, immersing the EHCPP-BDB polymer in 10% NaOH resulted in a 30% increase in the weight of the network. This is because the dynamically reversible boron oxygen bond is hydrolyzed into boric acid in an aqueous solution, which then forms borate in the presence of sodium hydroxide.

#### 3.2.2. Schiff Bases

**Scheme 15:**
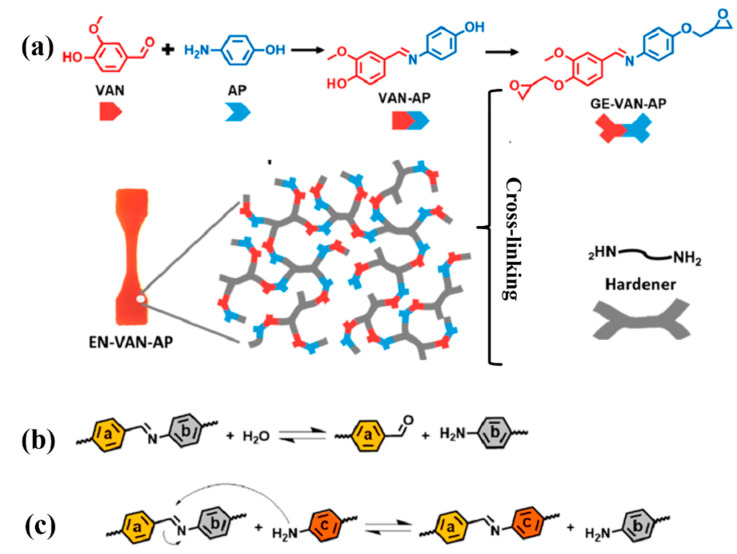
Synthesis of GE-VAN-AP and cured EN-VAN-AP (**a**), dissociative mechanism (**b**), and associative mechanism (**c**) of imine bond.

**Figure 9:**
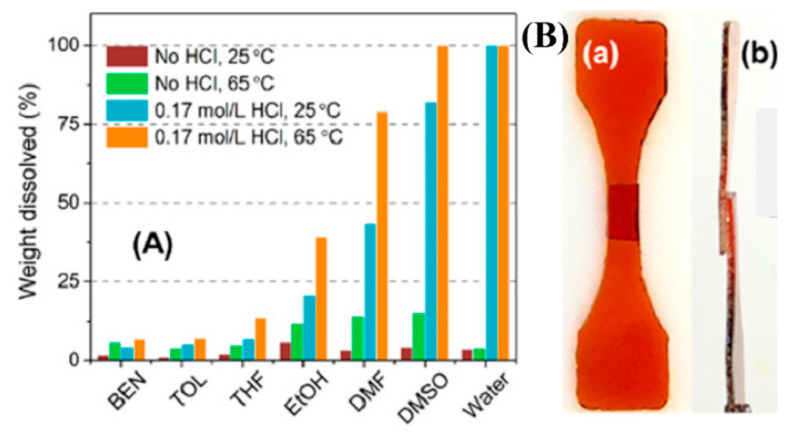
(**A**) Effects of solvent, HCl concentration, and temperature on the depolymerization and solubility of EN-VAN-AP. (**B**) Weldability of EN-VAN-AP: (**a**) Two thermoset films were overlapped and welded at 120 °C for 4 h, (**b**) Side view of the welded sample. Reproduced with permission from ref. [116]. Copyright 2018 American Chemical Society.

A Schiff base refers to a compound with the structure of R_2_C=NR′(R′H) formed by the reaction between an aldehyde and amine group. A Schiff base provides a promising process for the design of recyclable/degradable bio-based epoxy resins due to its three dynamic reactions, including imine–amine exchange (transamination), imine–imine exchange (imine metathesis), and imine condensation/hydrolysis [113,114,115,116,117,118,119,120,121,122,123,124]. Unlike transesterification, there is no need for metal catalysts during reprocessing, reducing the complexity of the reprocessing process. For example, M. Abu-Omar et al. synthesized the imine-embedded bisphenol intermediate of VAN-AP used to prepare the epoxy monomer (GE-VAN-AP) from vanillin and aminophenol (Scheme 15a) [116]. The cured epoxy thermosetting resin with diamine can be soluble in water or organic solvents under mild conditions (Figure 9A), which is conducive to achieving molecular closed-loop recyclability, and the recycled resin can maintain the original properties, and the dissociative mechanism is shown in Scheme 15b. Through the imine–amine exchange mechanism (Scheme 15c), the cured GE-VAN-AP is weldable and repairable at a sufficient temperature, as shown in Figure 9B. In addition, the cured resin has properties comparable to conventional high-performance thermosetting materials made of bisphenol A.

**Figure 10:**
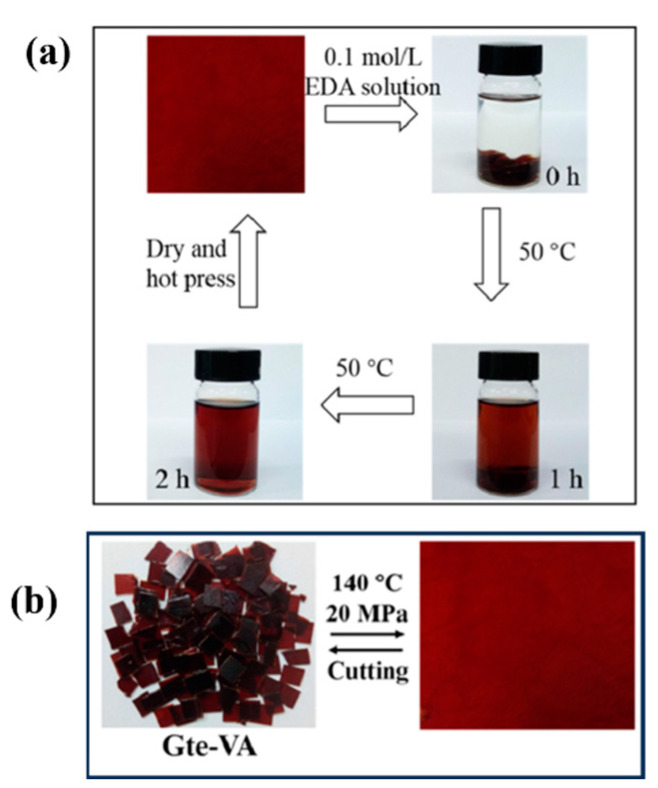
(**a**) Recycling process of Gte-VA in 0.1 mol·L^−1^ ethylenediamine solution (solvent: DMF), (**b**) digital photos of Gte-VA through cutting and compression molding. Reproduced with permission from ref. [119]. Copyright 2021 American Chemical Society.

**Figure 11:**
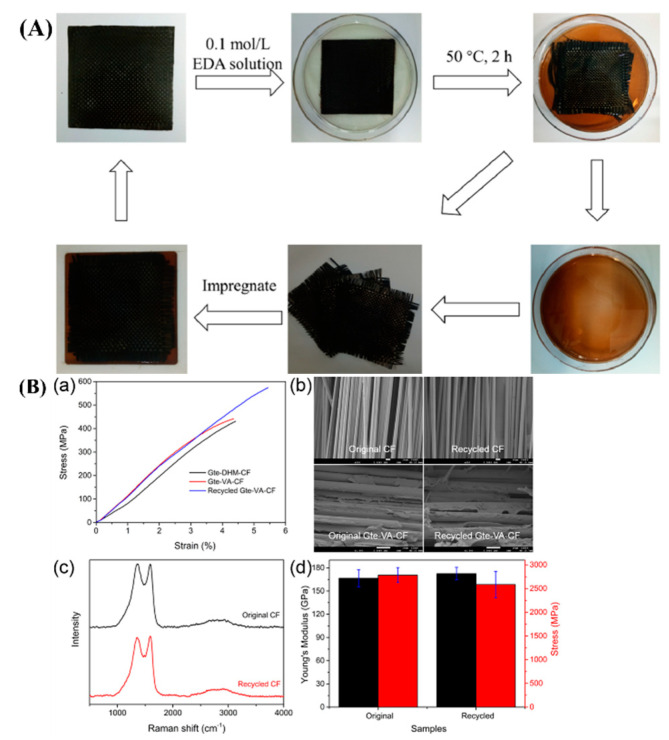
(**A**) Recycling of Gte-VA-CF in 0.1 mol·L^−1^ ethylenediamine solution (solvent: DMF), (**B**) (a) Stress-strain curves of Gte-VA-CF, and recycled Gte-VA-CF, (b) SEM for original CF and recycled CF and cross-sectional views of original Gte-VA-CF and recycled Gte-VA-CF, (c) Raman spectra of original CF and recycled CF, (d) Histogram of Young’s modulus and stress of original and recycled CF. Reproduced with permission from ref. [119]. Copyright 2021 American Chemical Society.

Epoxy curing agents containing a Schiff base structure can also be utilized to prepare polymer network structures with recyclable properties [119,120]. For example, Zeng and co-workers also synthesized VAN-AP and acted as a curing agent for glycerol triglycidyl ether (Gte) to form a polymer network structure (Gte-VA) embedded in the Schiff base structure [119]. The Young’s modulus and tensile strength of the Get-VA polymer network structure are close to the value of the amine-cured bisphenol A diglycidyl ether. The Get-VA polymer network structure has good degradation properties and reprocessing ability due to the embedding of Schiff base structures (Figure 10). In addition, Get-VA can also be used in the matrix of carbon fiber (CF) composite material. The carbon fiber in the composite material can be recycled without damage, and the recombination of the recycled carbon fiber and the degraded resin matrix has similar properties to the original composite material (Figure 11).

**Scheme 16:**
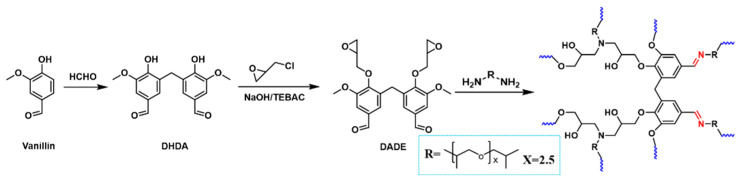
Synthesis route of the epoxy monomer of DADE containing aldehyde group from vanillin and curing mechanism of polyimine-epoxy resin.

**Figure 12:**
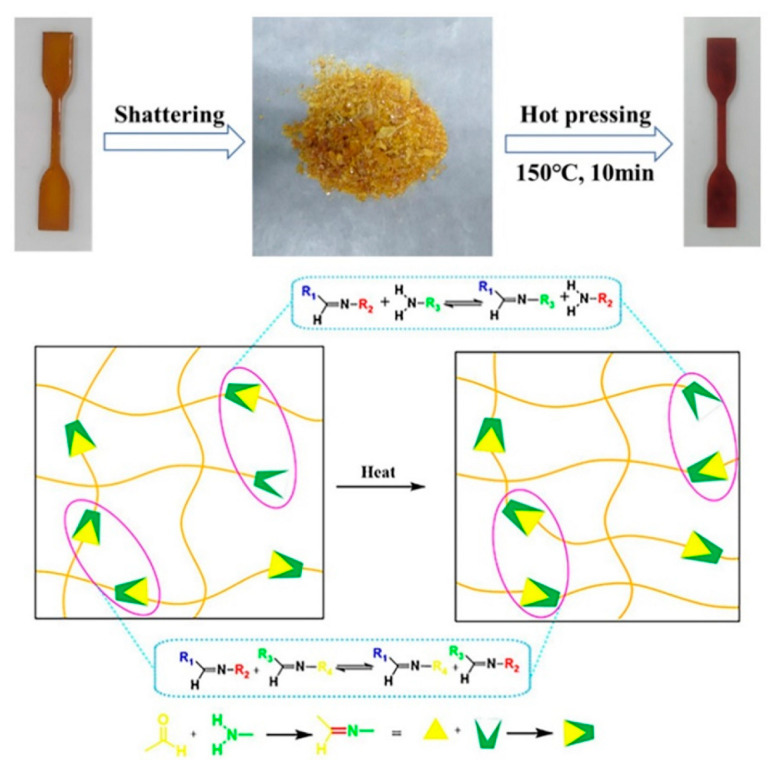
Reprocessing of DADE-D230 in a hot pressing and reprocessable mechanism of DADE-D230. Reproduced with permission from ref. [121]. Copyright 2020 Elsevier.

The introduction of Schiff base into polymer networks not only solves the degradability of bio-based epoxy but also improves the mechanical properties. And the Schiff base structure can be formed in situ between the epoxy monomer and the curing agent during curing. For example, Liu et al. also synthesized an epoxy monomer of DADE containing an aldehyde group from vanillin and cured it with a curing agent containing two primary amines group (D230) to form a polymer network (DADE-D230) containing Schiff base structure in situ (Scheme 16) [121]. This polymer network has a higher *T*_g_ (106 °C) and tensile strength (57.4 MPa) than the bisphenol A-based epoxy resin (98 °C, 45.1 MPa). The shattered polymer was remodeled after hot pressing at 150 °C for 10 min (Figure 12), and the reprocessed sample has comparable properties with the conventional bisphenol A-based epoxy resin. And the mechanisms involved in the reprocessing process are illustrated in Figure 12, including imine-amine exchange (via the excess amine in the network) and imine-imine exchange.

In addition, the introduction of Schiff base structures usually functionalizes polymer materials, such as flame-retardant and antibacterial properties [122,123,124]. At high temperature, the internal -CH=N- bonds of the bio-based epoxy-containing imine bonds will be rearranged and cyclized, resulting in nitrogen-containing six-membered heterocyclic compounds [71,90,91]. These heterocyclic compounds can make polymer chains self-crosslink, thus enhancing the char-forming ability of materials and improving the flame-retardant properties of materials. It provides a new possibility for degradable and flame-retardant epoxy resin. This category is described in detail in the antibacterial and flame-retardant functionalization Section.

#### 3.2.3. Acetal Structure

Aldehydes or ketones react with polyols under acidic conditions to form an acetal structure that is stable under alkaline conditions, but decomposes into the original aldehydes (ketones) and alcohols under acidic conditions. Therefore, the introduction of acetal structure into the epoxy resin network makes the epoxy resin degradable [125,126,127]. Moreover, the resin introducing this structure has excellent thermal and mechanical properties due to the high rigidity of the acetal structure.

**Figure 13:**
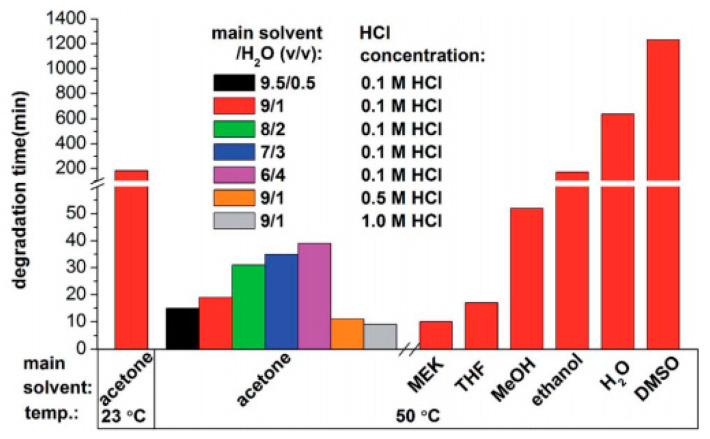
The degradation times (completely dissolved in acid solutions) under different conditions. Reproduced with permission from ref. [126]. Copyright 2019 The Royal Society of Chemistry.

**Scheme 17:**
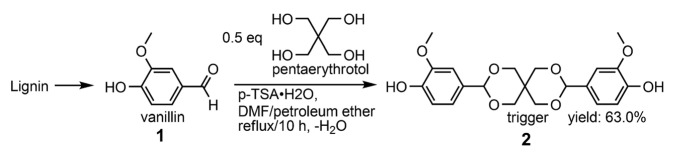
Synthetic route to spiro diacetal triggered from a renewable resource.

**Scheme 18:**
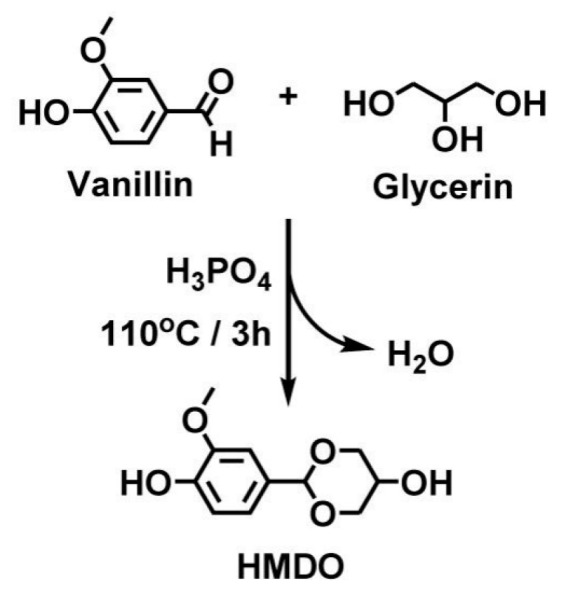
Synthetic route of HMDO.

**Scheme 19:**
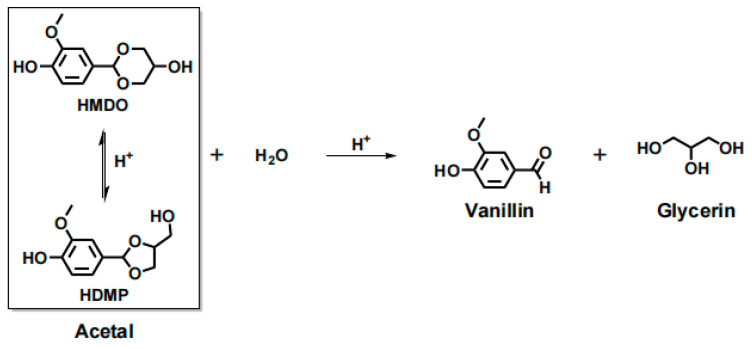
Hydrolysis mechanism of HMDO.

**Figure 14:**
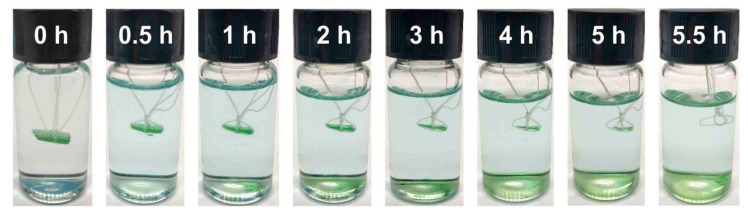
Chemical degradability of the HMDO-based thermosetting plastic. Reproduced with permission from ref. [127]. Copyright 2019 The Royal Society of Chemistry.

For example, Songqi Ma et al. used the spiro diacetal structure to prepare recyclable high-performance bio-based epoxy resin from vanillin. The synthetic route is shown in Scheme 17 [126]. The related epoxy resin cured with isophorondiamine exhibited outstanding degradability. As shown in Figure 13, the epoxy resin can be completely dissolved in 1 M HCl (acetone/H_2_O = 9/1, *v*/*v*) solution within 183 min at 23 °C. At 50 °C, the degradation time is reduced to 19 min, and the degradation time can be achieved in 11 min and 9 min, respectively, in solutions with concentrations of 0.5 M and 1 M HCl. The tensile strength of the vanillin-based CF composite is 731 MPa, while that of the BPA-based CF composite is 661 MPa. It is worth noting that the resin in the vanillin-based epoxy matrix composite can be completely dissolved in the 1 M HCl solution within 30 min, and maintain its carbon fiber properties. Similarly, the same authors synthesized a bisphenol of HMDO containing acetal from natural resources of vanillin and glycerol through solvent-free acetalization as in Scheme 18 [127]. HMDO can be degraded to non-toxic vanillin and glycerol very quickly under mildly acidic conditions similar to the acidity and temperature of human gastric juice in the human stomach (Scheme 19). Therefore, the obtained epoxy resins possess outstanding degradability, which facilitates the recyclability of the epoxy resin. An epoxy thermoset of DGHMDO-DDM sample with dimensions of 8 mm × 8 mm × 1 mm was completely degraded after being immersed in 0.1 M HCl acetone/water (9/1, *v*/*v*) solution at 50 °C for 5.5 h (Figure 14). In addition, compared to the commercial counterpart based on bisphenol A (BPA), DGHMDO-DDM showed higher mechanical properties and comparable thermal properties.

#### 3.2.4. Diels–Alder Addition Structures

**Scheme 20:**
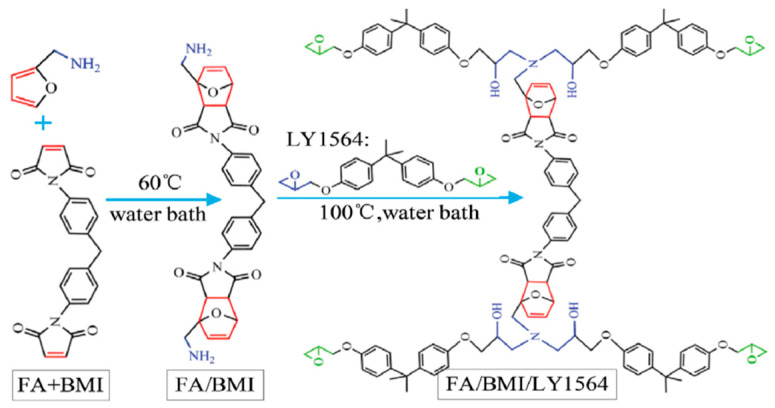
Synthesis of the self-healable resin system, including Diels–Alder (DA) structure.

**Scheme 21:**
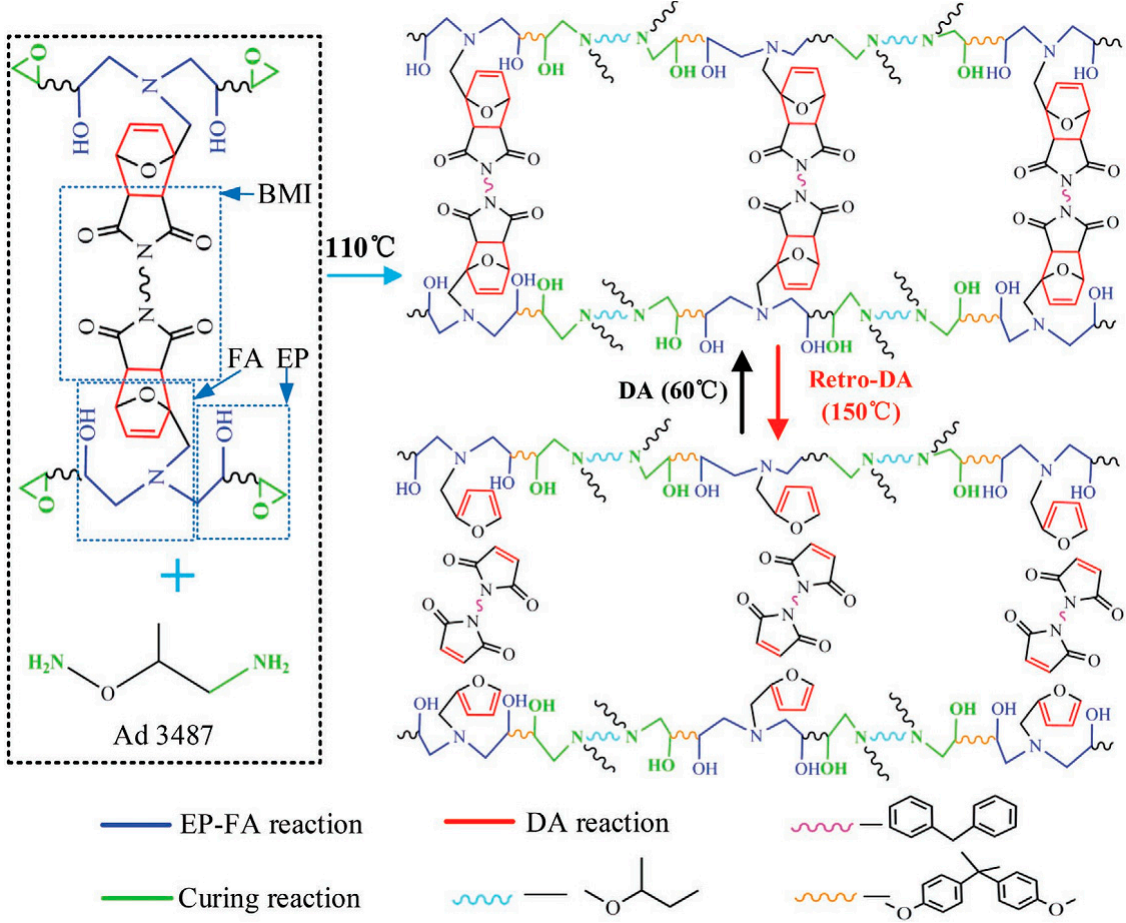
Mechanism of the self-healing process.

The D-A reaction (Diels–Alder reaction) occurs between an electron-rich conjugated diene and an electron-poor electrophilic diene, generating a cyclohexene adduct through a [4 + 2] cycloaddition reaction that occurs at a lower temperature (50–80 °C) and has the advantage of mild reaction conditions, catalyst-free requirement, and producing no byproducts in the reaction. The reverse reaction of the D-A reaction, retro-D-A reaction, occurs at higher temperatures (110–140 °C) to form the starting material adiene and conjugated diene [128,129,130,131,132,133,134]. Therefore, the introduction of the D-A addition structures into the thermosetting resin gives the material the self-healing and recyclable ability to make it sustainable. Furan derivatives and dimaleimide are often used as electron-rich and electron-poor feedstocks, respectively, in the design of bio-based thermosetting resins containing D-A structures. For example, Liu et al. Liu et al. prepared compounds containing D-A structures from furan and bismaleimide and grafted them onto a commercial epoxy monomer (as shown in Scheme 20) [131]. The results showed that the cured epoxy resin can completely self-heal its cracks. The resin is combined with unidirectional fiberglass cloth to form a fiber-reinforced plastic (FRP) material. Under the appropriate temperature stimulation, the resin matrix of FRP can also completely self-heal the crack through the dissociation and reassociation of D-A bonds (Scheme 21), and the mechanical properties of FRP after two self-healing are close to that of the original composite material. Therefore, the introduction of the D-A bond into polymer networks is a promising route for synthesizing epoxy resin with self-healing and recyclable properties.

#### 3.2.5. Disulfide Bonds

**Figure 15:**
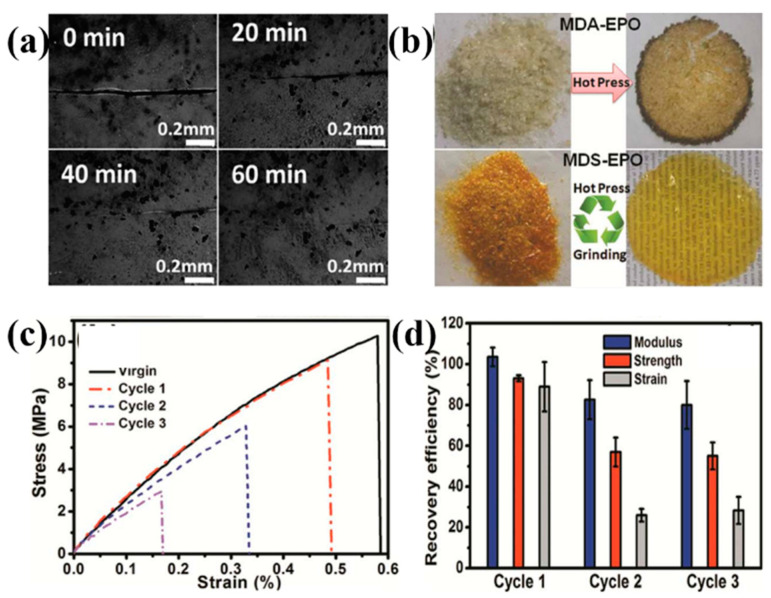
(**a**) Optical microscopic images of cracked MDS-EPO treated at 100 °C for various times, (**b**) reprocessing of MDA-EPO and MDS-EPO through a hot press at 100 °C for 1 h, (**c**) typical stress–strain curves and virgin and reprocessed MDS-EPO, (**d**) mechanical recovery efficiency of reprocessed MDS-EPO. Reproduced with permission from ref. [138]. Copyright 2017 Wiley-VCH.

The dynamic reaction of disulfide bonds involves multiple mechanisms. When the external force mechanically breaks the disulfide bond and produces the thiol radical, which can quickly exchange with other disulfide bonds, it achieves the self-healing ability and reprocessability of the cross-linked network. The dissociation nature of the disulfide bond provides a new solution for the degradation of the epoxy resin under reducing or alkaline conditions. Using dynamic disulfide bonds, many polymers with reprocessable and degradable properties have been synthesized and reported [135,136,137,138,139,140,141]. Wang et al. used traditional aromatic diamine and structurally similar aromatic diamine containing dynamic disulfide bonds as curing agents, respectively, to cure isosorbide-derived epoxy monomers to form polymer networks. Experimental studies showed that the epoxy resin-containing dynamic disulfide bond (MDS-EPO) exhibited comparable thermomechanical properties to the epoxy resin cured by the traditional curing agent (MDA-EPO). The cracked MDS-EPO was heated at 100 °C, above its *T*_g_, the crack gradually disappeared, and the crack was completely repaired after heating for 60 min (Figure 15a). The MDS-EPO broken into a powder can also be reprocessed into a uniform and transparent film at 100 °C for 60 min (Figure 15b). Moreover, the sample after one cycle of reprocessing has similar mechanical properties to the original sample, and the recovery efficiency of mechanical properties after the two and three cycles of reprocessing remained more than 80% (Figure 15c,d). The contrast of MDA-EPO broken into a powder had only physical adhesion without mechanical properties under the same thermal pressure conditions (Figure 15b) [138].

**Scheme 22:**
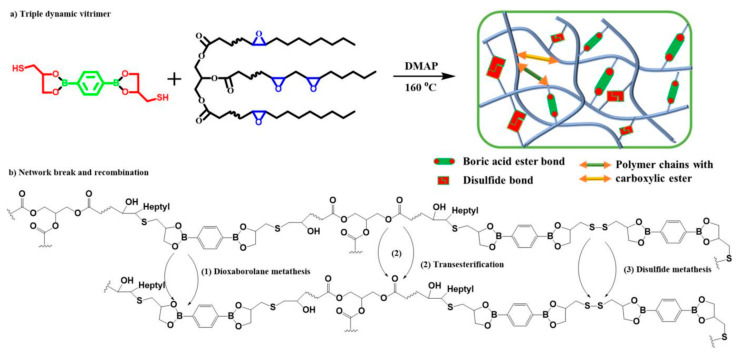
The reaction mechanism of cross-linked bio-based epoxy resins.

**Figure 16:**
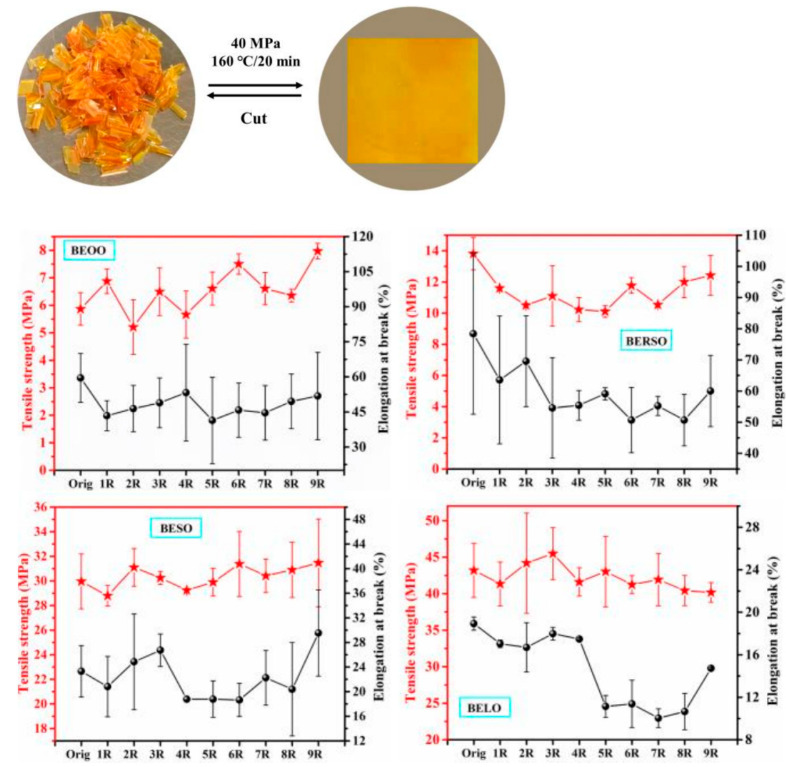
Reprocessing of cross-linked bio-based epoxy resins and mechanical properties of the reprocessed epoxy resins. Reproduced with permission from ref. [139]. Copyright 2021 The Royal Society of Chemistry.

The self-healing and reprocessing of the polymer networks containing a dynamic bond are generally slow, and the synergetic incorporation of multiple dynamic bonds can accelerate the stress relaxation of the polymer chains, enabling the polymer chains to complete the self-heal quickly at a moderate temperature at short period [139,140,141]. For example, Wang and co-workers used BDB as a curing agent to cure epoxidized soybean oil (ESO), epoxidized linseed oil (ELO), epoxidized rubber seed oil (ERSO), and epoxidized olive oil (EOO), respectively, to prepare bio-based epoxy resin, which contains three dynamic bonds including borate ester bond, ester bonds, and disulfide bonds (Scheme 22) [139]. These samples were cut into small pieces and remolded by hot-pressing at 160 °C and 40 MPa for 20 min to obtain the reprocessed samples, which maintain the original integrity and color (Figure 16), and the mechanical properties of the recycled samples are maintained even after nine times of cutting/recycling (Figure 16). These samples also have good self-healing properties. In addition, the introduction of a tricyclic dioxaborolane rigid structure improves the mechanical properties of vegetable oil-based epoxy resins, and the introduction of dynamic bonds gives the polymer self-healing and reprocessing properties. This example opens a new dimension to the tuning of polymer performance through multiple dynamic bonds.

### 3.3. Shape Memory

**Scheme 23:**
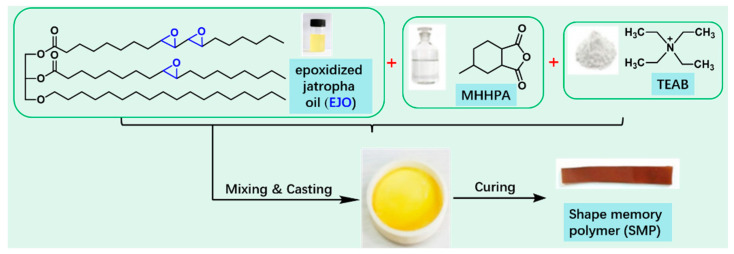
Schematic view of the preparation of jatropha oil-based shape memory polymer (SPM).

**Figure 17:**
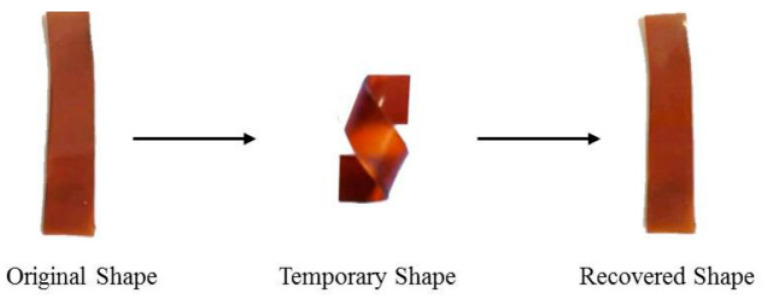
A demonstration of the shape memory recovery behaviors of EJO/MHHPA polymers reported in this paper. Reproduced with permission from ref. [145]. Copyright 2021 MDPI.

**Scheme 24:**
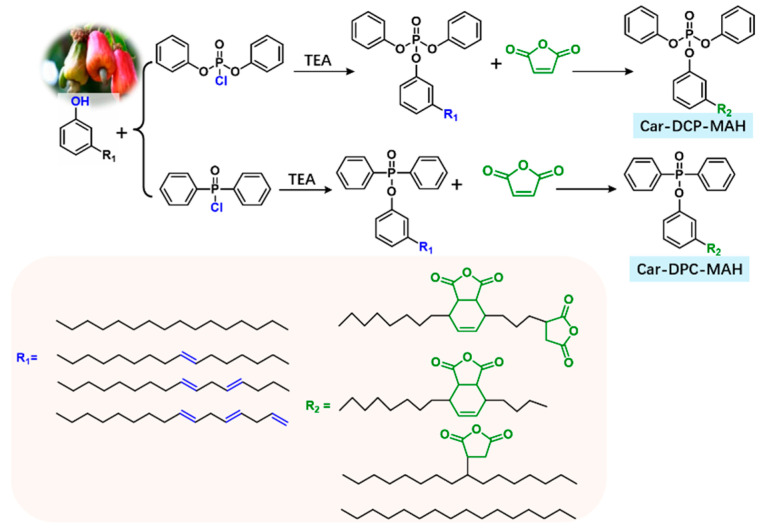
Synthetic processes of cardanol-based curing agents (Car-DCP-MAH and Car-DPC-MAH).

Shape memory polymers (SMPs) are materials that can be deformed into their shape under a certain condition (usually with external mechanical stress), and can recover to their permanent shape by applying external stimuli. Bio-based SMPs are primarily thermo-responsive, and their shape change is generally achieved by thermal stimulation [142,143,144,145,146,147,148,149,150,151,152,153,154] and they possess a transition temperature. Above the transition temperature, chain migration is activated and deformation occurs under external stress. Upon cooling with the load, the shape of the deformation is fixed. When heated to above transition temperature, it can be recovered to a permanent shape due to the entropy nature of the shape change. Because the long and flexible fat chain contributes to the chain segment migration, bio-based SMPs are typically made from bio-based feedstocks that contain flexible structures, such as vegetable oil, jatropha oil, and castor oil. For example, Aung and co-workers prepared epoxy polymers (SMPs) from jatropha oil (Scheme 23), which are in a thermally stable state at 150 °C. Above the *T*_g_ temperature, the polymers are in a rubbery state that is easily deformed, and they can return to their original shapes after cooling, and they have a good ability to recover their shapes (Figure 17) [145]. Cardanol is also often used in the preparation of bio-based epoxy polymers with shape memory due to its internal phenolic hydroxyl group, C=C carbon bonds, and long alkyl chain structure. Wang and co-workers synthesized two cardanol-derived curing agents (Car-DCP-MAH and Car-DPC-MAH, Scheme 24) and used them to cure DGEBA. The DGEBA/Car-DCP-MAH and DGEBA/Car-DPC-MAH epoxy thermosets not only have good shape memory (shape recovery efficiency of 98.6% and 96.3%, respectively), but also have high flame retardancy (LOI values of 29% and 28%, respectively, and a flame-retardant rating of V-0) [146].

**Figure 18:**
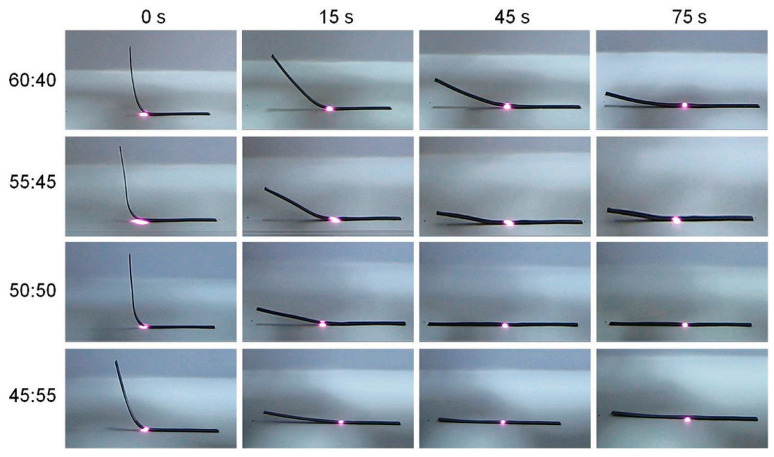
NIR laser-induced shape recovery of V-fa/ECO copolymers with different percent weight ratios of V-fa:ECO. Reproduced with permission from ref. [148]. Copyright 2020 Elsevier.

Thermal stimulation can be achieved not only by direct heating but also indirectly by light-converted heat [148,149]. The traditional photo-responsive materials are realized by doping photothermal fillers, such as black pigment, graphene, and nanosilver. However, photothermal fillers can aggregate in polymers and reduce the mechanical properties of the materials [149]. Therefore, finding photothermal groups from bio-based feedstocks and introducing them into the epoxy polymer network can improve this defect very well. A commonly used bio-based raw material is furfural [148,149]. For example, Rimdusit et al. reported epoxy polymers with near-infrared light-responsive shape memory function but without any photothermal fillers in the material [148]. A benzoxazine/epoxy copolymer (V-fa/ECO) with shape memory ability was made from furfurylamine. Within 30 s of near-infrared irradiation (laser power density of 2.5 W/cm^2^), the temperature of the copolymer increased from room temperature to 100 °C, resulting in a photothermal effect inducing shape recovery (Figure 18). This is due to the highly conjugated π-bonds in the furfuryl amine, which endow the furfural-based materials with excellent light absorption [148]. In addition, the aldehyde group in furfural is easily oxidized and exhibits a dark brown color making it a good light absorber [149].

**Figure 19:**
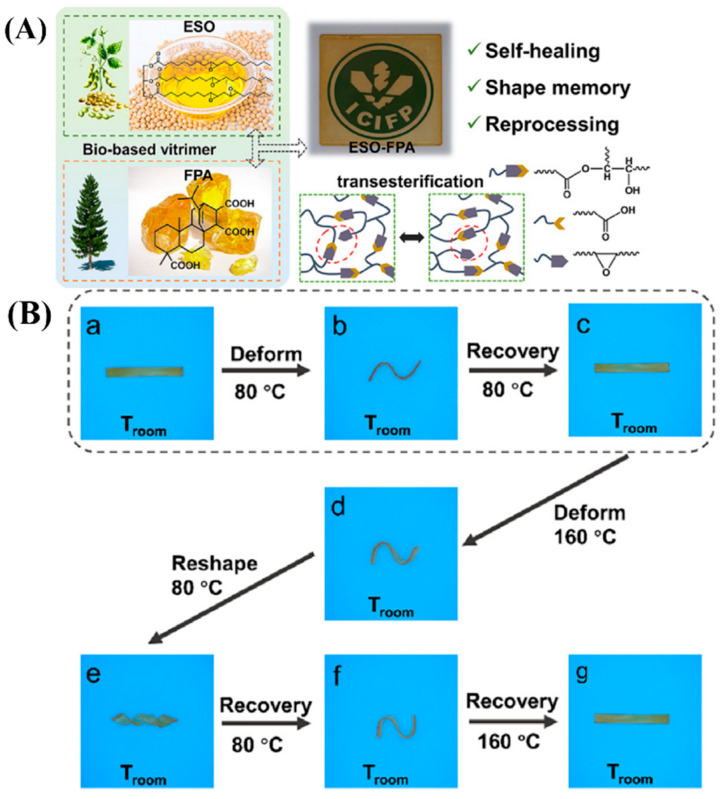
Chemical structure of bio-based epoxy monomer of ESO and bio-based curing agent of FPA, and topological network rearrangements via exchange reactions in ESO-FPA (**A**), and the triple-shape memory of ESO-FPA (**B**; (**a**–**c**) double shape memory of the vitrimer using the glass transition temperature to fix in an ‘N’-shape, and their recovery upon heating, (**c**–**g**) triple-shape memory of the vitrimer using the topology freezing transition temperature to fix the ‘N’-shape, and the glass transition temperature to fix the spiral shape, and their sequential recovery upon heating.). Reproduced with permission from ref. [151]. Copyright 2020 Elsevier.

**Figure 20:**
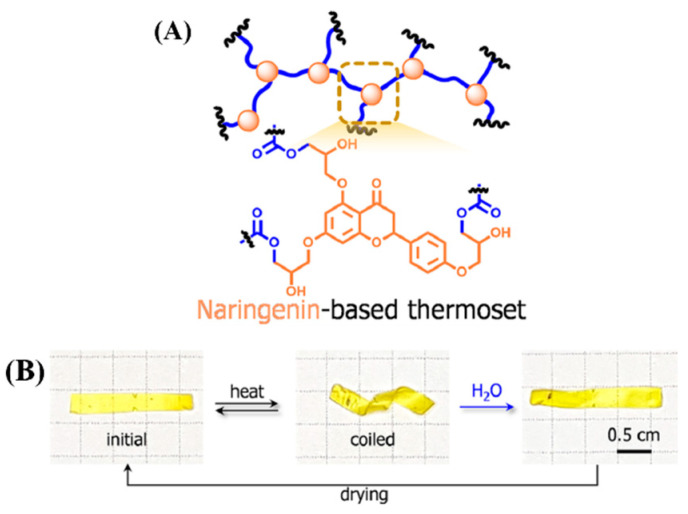
The polymer network of naringenin-based thermoset (**A**), and the dual responsive behavior using water and heat (**B**). Reproduced with permission from ref. [152]. Copyright 2019 American Chemical Society.

However, all of these materials above can only hold a single permanent shape and cannot be reconfigured once formed. The introduction of dynamic covalent bonds enables the bio-based shape memory thermosetting network to be topologically arranged through the exchange of dynamic bonds, and the permanent shape of the material can be further reconfigured [150,151,152,153]. Other than the transition temperature, the SMPs with reconfigured permanent shapes possess the topology freezing transition temperature, above which the dynamic covalent bonds are activated and the networks are topologically arranged through bond exchange, forming new permanent shapes. In addition, the introduction of dynamic bonds also provides a new idea for the development of recyclable and repairable shape memory epoxy polymers. The dynamic bonds commonly introduced are the Diels–Alder bond, disulfide bond, and ester bond [134,150,151,152,153]. For example, Xu and co-workers used ESO and rosin derivative fumarhippoic acid (FPA) as raw materials to synthesize a new fully bio-based vitrimer (ESO-FPA) with *T*_g_ higher than room temperature (Figure 19A). This polymer network can realize heat-induced triple-shape memory well (Figure 19B). Firstly, the shape memory of the ESO-FPA vitrimer based on the transition temperature was confirmed, as shown in Figure 19B. In an oven at 80 °C, the permanent rectangular sample was heated under external force so that it took a temporary “N” shape, which then was cooled and fixed. The temporary “N”-shaped sample can be recovered to its original permanent shape when heated at 80 °C. Then, the shape memory of the ESO-FPA vitrimer based on the transesterification was explored, as shown in Figure 19B. The permanent rectangular sample was heated at 160 °C (above the topology freezing transition temperature) under an external force to achieve a deformed “N” shape, which then was cooled and fixed. The deformed “N” as a new permanent shape is then deformed again at 80 °C under external stress, forming a temporary helical shape and being fixed. Since 80 °C is higher than the transition temperature and lower than the topology freezing transition temperature, the formation of temporary helical shapes was achieved through molecular chain movement. The temporary helical-shaped sample can be recovered to the new permanent “N” shape when heated at 80 °C. Finally, when heated to 160 °C, the ESO-FPA vitrimer with the “N” shape returned to its original permanent rectangular shape under extra stress. In addition, this new fully bio-based vitrimer also has good self-healing and reprocessability due to transesterification reaction [151]. Similarly, Cheng and co-workers prepared a novel bio-based recyclable phase change material (PCM) denoted as DGEM-18/FA/MA/xBW, which is a reversible cross-linked network based on epoxide ring-opening and Diels-Alder reactions. The D-A and retro-D-A reactions confer excellent triple-shape memory to the material and also provide the recyclability of the cross-linking networks [134]. Moreover, Kim et al. prepared a new type of sustainable thermosetting material using naringenin [152]. Then, it was cured by oxidized poly(ethylene glycol)-acid with functional groups of carboxylic acid to produce an epoxy network (Figure 20A). Transesterification in the network not only gives the material recyclability, but also realizes its heat-induced shape memory function. In addition, the shape change can be driven by water, and its coiled shape can be programmed on the *T*_g_, deformed after wetting, and restored to the initial state after short drying, realizing the dual-response shape memory of heat and water (Figure 20B) [152]. Further, the synergistic effect of multiple dynamic covalent bonds also provides a new way to prepare bio-based and shape memory epoxy resins with reprocessability, repairability, and recyclability. Mija et al. prepared epoxy thermosetting resin with dynamic disulfide covalent bonds and transesterification synergies using 2,2′-dithiobenzoic acid as a hardener. The obtained epoxy thermosetting has excellent shape memory ability, and the synergistic effect of internal covalent bonds ensures the reprocessability, repairability, and recyclability of the material [153]. This provides a new experience for sustainable epoxy thermosetting materials with shape memory.

### 3.4. Antibacterial

**Figure 21:**
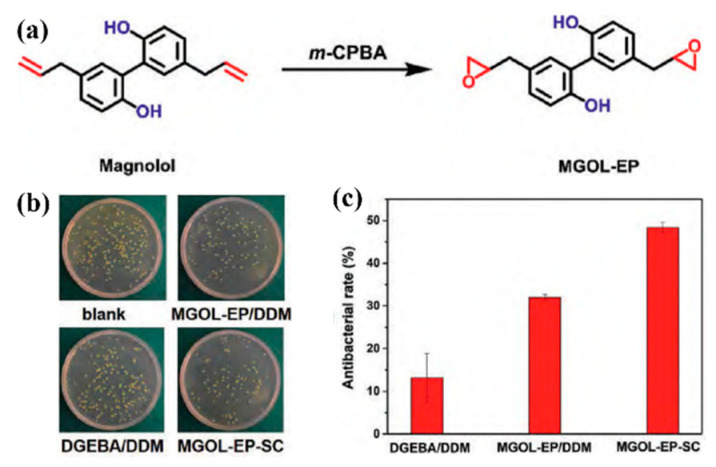
(**a**) Synthesis of epoxy monomer of MGOL-EP. (**b**) Staphylococcus aureus colonies survived on the nutrient agar plates after treatment with blank, DGEBA/DDM, MGOL-EP/DDM, and MGOL-EP-SC. (**c**) The antibacterial rates against Staphylococcus aureus of various samples. Reproduced with permission from ref. [157]. Copyright 2022 Elsevier.

Antibacterial materials usually achieve their bactericidal effect through the release of antibacterial components such as metal nanoparticles, antibiotics, and other biocides, loaded or embedded in the resin matrix. However, with the consumption of biocides, the antibacterial effect gradually disappears, making it difficult to maintain a long-lasting bactericidal effect [36,155,156,157,158,159,160,161,162,163,164]. By directly functionalizing the biocidal units into the molecular chains, a longer-lasting antibacterial effect can be achieved [156,157,158,159,160]. For example, phenolic compounds (magnolol, eugenol, vanillin, protocatechuic acid, etc.) have natural antibacterial activities and are often used as raw materials for antibacterial materials [157,158,159,160]. They act on bacterial cells through various mechanisms, such as interacting with protein and bacterial cell walls, changing the membrane permeability, inhibiting the synthesis of nucleic acid, or damaging DNA [161]. On the one hand, antibacterial units can be functionalized into the main chain. Magnolol, as a natural product containing both phenolic and allylic groups, has strong antibacterial activity. Cao et al. synthesized a bio-based epoxy monomer (MGOL-EP) from natural magnolol (Figure 21a). A fully bio-based and high-performance epoxy thermosetting resin (MGOL-EP-SC) was obtained by self-curing, and its killing rate against *S. aureus* reached 48.4% (Figure 21b,c). The antibacterial property was attributed to the mechanism that MGOL-EP-SC resin can damage the cell walls, causing increased membrane permeability, leakage of cell components, and inhibition of *S. aureus* growth [157]. On the other hand, antibacterial units can also be functionalized into the side chains [158,159,160]. For example, Modjinou et al. designed an epoxidized eugenol network with antioxidant and antibacterial properties using eugenol derivative (named epoxy eugenol, EE), and the combination of eugenol derivative with a phenolic group and the addition of 70 wt% eugenol derivative can reduce the bacterial adhesion of both *S. aureus* and *E. coli* by more than 90% [159].

**Scheme 25:**
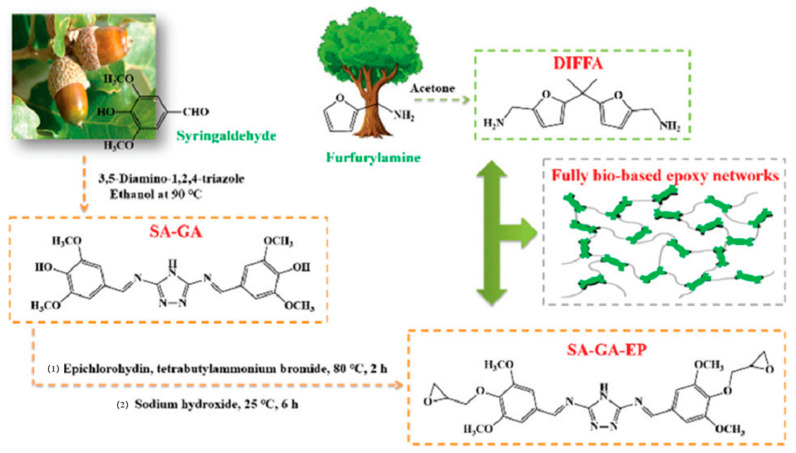
Synthetic route to the fully bio-based epoxy thermoset from syringaldehyde-derived epoxy monomer (SA-GA-EP) cured by a furan-derived amine (DIFFA).

**Figure 22:**
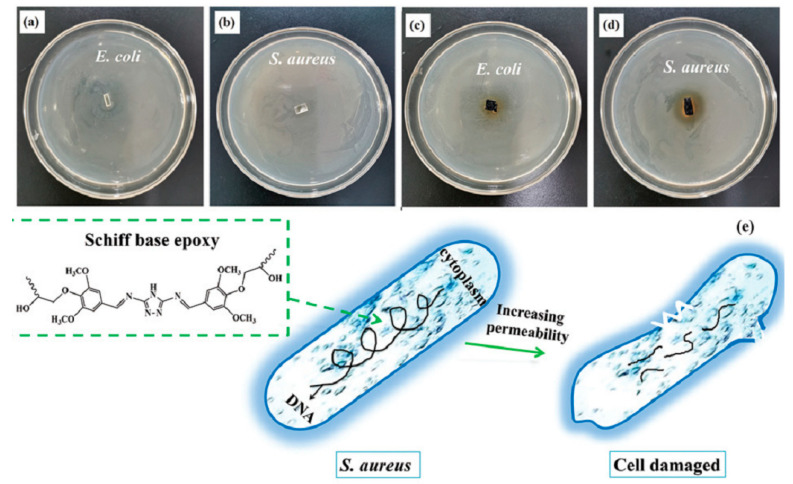
Antibacterial activities of (**a**,**b**) DGEBA/DDM and (**c**,**d**) SA-GA-EP/DIFFA against *E. coli* and *S. aureus*; and (**e**) antibacterial mechanism of SA-GA-EP/DIFFA. Reproduced with permission from ref. [155]. Copyright 2020 The Royal Society of Chemistry.

In addition, new antibacterial groups can be designed by modifying bio-based monomers. The most studied are Schiff bases formed by aldehydes/ketones reacting with amines (imine bonds and hydrazone bonds) [155,162,163,164,165]. The antibacterial mechanism of Schiff bases may be related to the free electrons in the nitrogen atoms, which facilitates the antibacterial material to easily interact with the bacteria cell wall/membrane [166]. Tang et al. prepared a bio-based thermosetting material with high performance, high flame retardancy (limiting oxygen index 33.8%, UL-94 V-0 rating), and antibacterial properties. The Schiff bases in the network provided 100% intrinsic antibacterial efficiency of epoxy resin, and it had good antibacterial activity against *E. coli* and *S. aureus*. It is satisfactory that this material also has good reprocessability and degradability due to the embedding of a Schiff base structure [163]. However, fully bio-based epoxy materials with simultaneously high mechanical, thermal, and antibacterial properties have been rarely reported. Hu and co-workers obtained a fully bio-based epoxy thermosetting resin (SA-GA-EP/DIFFA) from syringaldehyde-derived epoxy monomer containing a Schiff base and curing agent of DIFFA synthesized from furylamine (Scheme 25) [155]. Because of the Schiff base structure, SA-GA-EP/DIFFA not only has good antibacterial activity against Gram-positive *S. aureus* (Figure 22), but also has a high *T*_g_ up to 204 °C, a flame-retardant rating of UL-94 V-0, and a high limiting oxygen index (LOI) up to 40.0%, which provides a new research avenue for the fully bio-based epoxy thermosets to become high-performance and antibacterial materials [155].

In order to assess the practical viability and competitiveness of bio-based alternatives in real applications. SA-GA-EP/DIFFA, MGOL-EP-SC, and MGOL-EP/DDM are taken as typical representatives, and their mechanical properties, thermal stability, antibacterial, and flame-retardant properties are summarized in Table 2 to compare with that of petroleum-based epoxy resin. It can be seen from Table 2 that the comprehensive performance of the SA-GA-EP/DIFFA, MGOL-EP-SC, and MGOL-EP/DDM is better than that of petroleum-based epoxy resins, so they are promising substitutes for a DGEBA-based thermoset in high-performance fire safe applications.

**Scheme 26:**
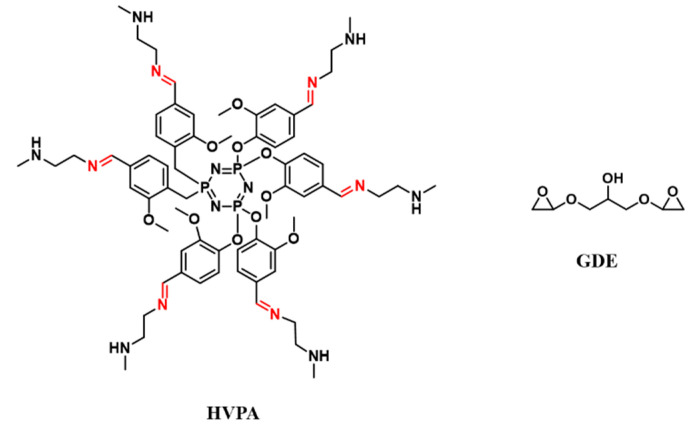
Chemical structure of HVPA and GDE.

**Figure 23:**
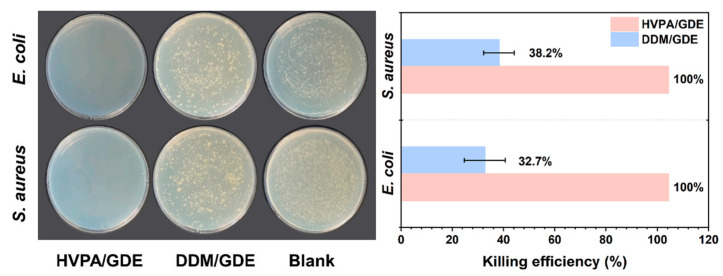
Antibacterial effect of HVPA/GDE epoxy film on *E. coil* and *S. aureus*. Reproduced with permission from ref. [163]. Copyright 2022 Elsevier.

**Scheme 27:**
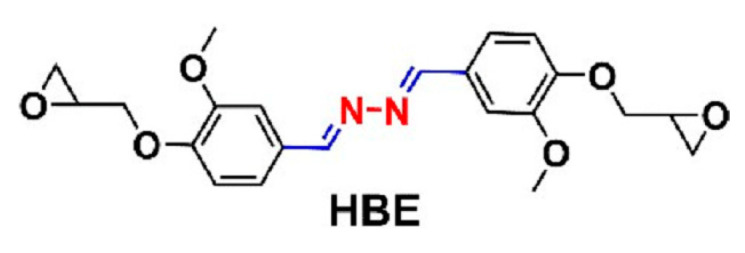
Chemical structure of HBE containing hydrazone group.

**Figure 24:**
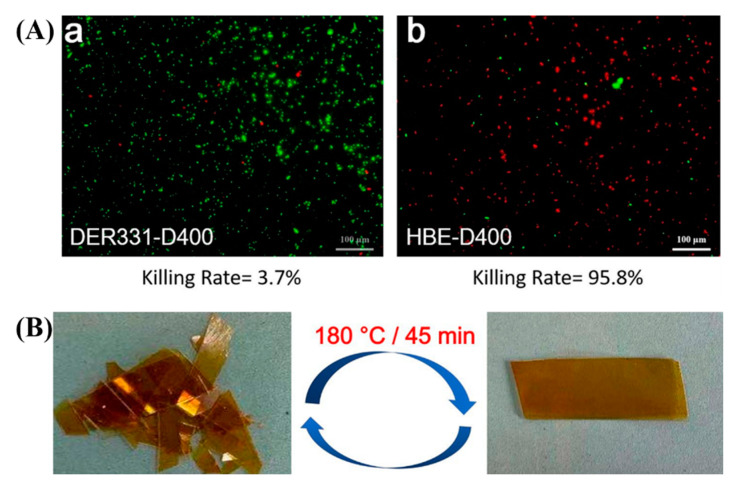
(**A**) Antibacterial activities of DER331-D400 (**a**) and HBE-D400 (**b**), (**B**) reprocessing of HBE-D400 (DER331 is the trade name of DGEBA). Reproduced with permission from ref. [164]. Copyright 2020 The Royal Society of Chemistry.

The manufacture of epoxy resin with flame retardancy, antibacterial properties, degradability, and high mechanical properties is difficult due to the contradiction between the high cross-linking densities required for high-performance and flame retardancy, and the mild and dynamic reversible conditions for reprocessability and degradability. Liu and coworkers prepared a bio-based epoxy resin (HVPA/GDE) by curing the bio-based epoxy of glycerol diglycidyl ether (GDE) with a novel vanillin-derived curing agent (HVPA)-containing Schiff base (Scheme 26). Due to the uniform distribution of dynamic imine bonds in each cross-linking bond, the material not only has excellent flame retardancy (LOI value of about 28.6%, UL-94 V-0 grade) and recyclability, but also the calculated kill rate of both Escherichia coli and Staphylococcus aureus is about 100% (Figure 23). In addition, HVPA/GDE has comparable mechanical properties to the epoxy resin formed by curing GDE with a DDM curing agent. This gives the epoxy resin an excellent balance of fast self-healing (100% within 8 min, no pressure), good shape memory, easy reprocessing (1.5 MPa, 3 min, 100 °C), mild degradation (acid hydrolysis), bacterial resistance, and acceptable mechanical properties [164]. Furthermore, the hydrazone group as an important Schiff base structure also has a good antibacterial effect. Xu et al. synthesized dihydrazone-containing epoxide monomers (HBE, Scheme 27) by condensation of vanillin and hydrazine hydrate, and prepared a novel dihydrazone-based covalent network by cross-linking. Due to the hydrazone bond and methoxy on vanillin, the killing rate of E. coli was as high as 95.8% (Figure 24A), and the dihydrazone covalent network still showed excellent stability and hydrazone exchange at about 100 °C, which gave it excellent mechanical properties and recyclability (Figure 24B) [165].

## 4. Conclusions, Challenges, and Opportunities

The raw material of bio-based epoxy resin is natural biomass, which has the advantages of being sustainable and environmentally friendly and is the most promising substitute for petroleum resources for the preparation of bio-based epoxy resin. Bio-based epoxy resins with different functions have their own structural characteristics. In this review, functional bio-based epoxy resins are classified according to their structural characteristics, and their synthesis is discussed. For example, the synthesis of flame-retardant epoxy resins is usually achieved by introducing flame-retardant elements (P, Si, etc.), or rigid and conjugated structures. For the preparation of recyclable/degradable/processable bio-based epoxy resins, dynamic covalent bonds including ester bonds, disulfide bonds, Schiff base structures, D-A addition structures, and acetal structures are usually introduced into the polymer networks. Moreover, the introduction of these dynamic covalent bonds is often used in the preparation of epoxy resins with shape memory. And the design of antibacterial bio-based epoxy resins is usually realized by introducing antibacterial units such as hydroxyl, Schiff base, and long alkyl chains. Then, the relationship between structure and performance was summarized and revealed. The purpose of this review is to provide guidelines for the synthesis of functional bio-based epoxy resins and stimulate the development of more types of functional bio-based epoxy resins. However, due to the different chemical reactions that can occur for biomass raw materials with different structures, a unified design and synthesis method cannot be given.

Functional bio-based epoxy resins improve their competitiveness because of their unique properties, but there are still many problems to be solved. For example, biomass feedstock has the characteristics of higher cost and smaller production scale. How to improve the separation and purification technology of biomass and synthesize bio-based epoxy resin with high purity and economic efficiency is a problem that needs to be solved at present. In addition, most bio-based epoxy monomers are solid, and are more difficult to process than DGEBA. For bio-based epoxy resins with recyclable/degradable/reprocessable properties or shape memory ability, mechanical properties are often deteriorated by the introduction of dynamic covalent bonds or flexible long chains. Therefore, seeking the balance between mechanical properties and functional properties is a problem that needs to be considered comprehensively in design and synthesis. However, flame-retardant epoxy resins containing aromatic rings with a high char yield usually have brittle lack. In addition, it should also be noted that high temperature may cause functional disappearance during processing. Multifunctional bio-based epoxy resins should be developed, and this review also lists several bio-based epoxy resins that are both flame-retardant and antibacterial, or both flame-retardant and recyclable. Furthermore, more new kinds of functions need to be developed to meet the needs of practical applications. And further research is necessary to determine how to increase the bio-based content of epoxy resins.

## Figures and Tables

**Table 1:** Summary of studies on phosphorus-containing (yellow part), phosphorus-free (blue part) flame-retardant epoxy resins and their counterpart of DGEBA (red part).

Epoxy Resin	Curing Agent	*T*_g_ (°C)	*T*_d5%_ (°C)	Char Yield (wt%)	UL-94	LOI (%)	Combustion Results	Ref.
TDBE	DDM	140	217	Y_700_ = 29.8	V-0	42.0	pHRR-69.3% THR-53.0%	[38]
DGEBDB/ DGEBA = 1/9	DDM	176.4	-	Y_800_ = 19.1	V-1	30.2	THR-11.9%	[39]
DGEBDB/ DGEBA = 2/8	DDM	169.2	-	Y_800_ = 21.2	V-0	32.4	THR-18.3%	[39]
VDE	DDM	129.6	248.8	Y_800_ = 28	V-0	34.5	-	[40]
VSE	DDM	176.1	254.9	Y_800_ = 34.5	V-0	38.7	-	[40]
TEBA	DDM	136	271	Y_700_ = 29.9	V-0	42.3	pHRR-67% THR-27%	[41]
MEP/ DGEBA = 8/2	DDM	147.1	314.3	-	V-0	27.5	pHRR-34.7% THR-27.0%	[42]
VAD-EP/VDP-EP = 8/2	D230	82.3	212.7	Y_800_ = 32.8	V-0	27.0	-	[43]
VAD-EP/VDP-EP = 7/3	D230	80.3	205.3	Y_800_ = 30.2	V-0	28.7	pHRR-47.9% THR-32.0%	[43]
EHEP	D230	122	270	Y_700_ = 39.0	V-0	31.0	pHRR-66% THR-65%	[44]
HECarCP	DDM	-	-	Y_800_ = 14.3	V-0	33.0	pHRR-63% THR-24%	[45]
BEEP/DGEBA = 2/8	DDM	138.7	332.2	Y_800_ = 23.9	V-0	27.5	pHRR-25.9% THR-40.2%	[46]
PPDEG-EP	DDM	-	203	Y_800_ = 30.47	V-0	32.1	THR-21.0%	[48]
GPEP	DDM	130.0	298.1	Y_800_ = 31.0	V-0	31.2	pHRR-77.7% THR-65%	[49]
BEU-EP	DDM	112.3	300.5	Y_700_ = 23.4	V-0	38.4	pHRR-84.9%	[50]
TEUP-EP	DDM	203.7	320.3	Y_800_ = 43.8	V-0	31.4	pHRR-63.1% THR-57.4%	[51]
EP1	DDM	183	340	Y_700_ = 53	V-0	31.4	-	[54]
EP2	DDM	214	353	Y_700_ = 58	V-0	32.8	-	[54]
MDE	DDM	178	361.4	Y_750_ = 28.3	V-0	44.9	pHRR-64.5% THR-59.2%	[56]
DGEM	DDS	279	402	Y_800_ = 42.8	V-0	-	pHRR-70% THR-26%	[58]
MTEP	DDS	326	377	Y_700_ = 52.1	V-0	-	pHRR-56.7%	[59]
RESEP	DDM	335	352.6	Y_800_ = 38.6	V-0	31.8	-	[60]
DGEFA	DDM	178.5	316.4	Y_700_ = 40.6	V-1	32.8	pHRR-39% THR-43%	[61]
HCA-EP	DDM	192.9	319.0	Y_700_ = 31.6	V-1	32.6	pHRR-60%	[62]
DGEC	DDS	300	324	Y_800_ = 50.15	V-0	-	pHRR-74.8% THR-59.4%	[63]
GV-EP	DDM	220	290.5	Y_800_ = 44.9	V-0	35.5	pHRR-86.4% THR-48.1%	[64]
DGEBA	DDM	187	368	Y_800_ = 12.8	no rating	22.6	pHRR = 646 W/g THR = 23.9 kJ/g	[64]
TVEP	difuran diamine	162.7	275.4	Y_800_ = 30.0	V-0	28.5	THR-52.6%	[65]
PH-ODA-EP	DDM	204.9	280.1	Y_700_ = 41.7	V-0	40.5	pHRR-92.9% THR-72.0%	[66]
DGED	DDM	205	335	Y_800_ = 42.9	V-0	31.6	-	[67]
DGEL	DDS	314.4	379	Y_700_ = 44.0	V-0	32.5	pHRR-74.5% THR-16.5%	[68]
DGEG	DDM	223	-	Y_800_ = 43.8	V-0	33.1	pHRR-36.9% THR-48.5%	[69]
DGEEA	DDM	220	262	Y_800_=38.5	V-0	40.0	pHRR-84.9% THR-70.1%	[70]
HMF-GU-EP	difuran diamine	222	283	Y_800_ = 55.8	V-0	39.5	pHRR-92.4% THR-73.1%	[71]
GSPZ-EP	DDM	187	331	Y_700_ = 42.3	V-0	-	pHRR-60.5% THR-54.4%	[72]
THMT-EP	DDS	300	394	Y_800_ = 44.8	V-0	35.4	pHRR-65.5% THR-29.3%	[73]
DGEBA	DDS	180	391	Y_800_ = 13.5	no rating	22.9	pHRR = 423 W/g THR = 27.3 kJ/g	[73]
Triazole-VA-EP	DDM	135	-	Y_800_ = 34.7	V-0	39.5	pHRR-82.3% THR-52.8%	[74]
TDBE	DDM	140	217	Y_700_ = 29.8	V-0	42.0	pHRR-69.3% THR-53.0%	[38]
DGEBDB/ DGEBA = 1/9	DDM	176.4	-	Y_800_ = 19.1	V-1	30.2	THR-11.9%	[39]
DGEBDB/ DGEBA = 2/8	DDM	169.2	-	Y_800_ = 21.2	V-0	32.4	THR-18.3%	[39]
VDE	DDM	129.6	248.8	Y_800_ = 28	V-0	34.5	-	[40]
VSE	DDM	176.1	254.9	Y_800_ = 34.5	V-0	38.7	-	[40]
TEBA	DDM	136	271	Y_700_ = 29.9	V-0	42.3	pHRR-67% THR-27%	[41]
MEP/ DGEBA = 8/2	DDM	147.1	314.3	-	V-0	27.5	pHRR-34.7% THR-27.0%	[42]
VAD-EP/VDP-EP = 8/2	D230	82.3	212.7	Y_800_ = 32.8	V-0	27.0	-	[43]
VAD-EP/ VDP-EP = 7/3	D230	80.3	205.3	Y_800_ = 30.2	V-0	28.7	pHRR-47.9% THR-32.0%	[43]
EHEP	D230	122	270	Y_700_ = 39.0	V-0	31.0	pHRR-66% THR-65%	[44]
HECarCP	DDM	-	-	Y_800_ = 14.3	V-0	33.0	pHRR-63% THR-24%	[45]
BEEP/DGEBA = 2/8	DDM	138.7	332.2	Y_800_ = 23.9	V-0	27.5	pHRR-25.9% THR-40.2%	[46]
PPDEG-EP	DDM	-	203	Y_800_ = 30.47	V-0	32.1	THR-21.0%	[48]
GPEP	DDM	130.0	298.1	Y_800_ = 31.0	V-0	31.2	pHRR-77.7% THR-65%	[49]
BEU-EP	DDM	112.3	300.5	Y_700_ = 23.4	V-0	38.4	pHRR-84.9%	[50]
TEUP-EP	DDM	203.7	320.3	Y_800_ = 43.8	V-0	31.4	pHRR-63.1% THR-57.4%	[51]
EP1	DDM	183	340	Y_700_ = 53	V-0	31.4	-	[54]
EP2	DDM	214	353	Y_700_ = 58	V-0	32.8	-	[54]
MDE	DDM	178	361.4	Y_750_ = 28.3	V-0	44.9	pHRR-64.5% THR-59.2%	[56]
DGEM	DDS	279	402	Y_800_ = 42.8	V-0	-	pHRR-70% THR-26%	[58]
MTEP	DDS	326	377	Y_700_ = 52.1	V-0	-	pHRR-56.7%	[59]
RESEP	DDM	335	352.6	Y_800_ = 38.6	V-0	31.8	-	[60]
DGEFA	DDM	178.5	316.4	Y_700_ = 40.6	V-1	32.8	pHRR-39% THR-43%	[61]
HCA-EP	DDM	192.9	319.0	Y_700_ = 31.6	V-1	32.6	pHRR-60%	[62]
DGEC	DDS	300	324	Y_800_ = 50.15	V-0	-	pHRR-74.8% THR-59.4%	[63]
GV-EP	DDM	220	290.5	Y_800_ = 44.9	V-0	35.5	pHRR-86.4% THR-48.1%	[64]
DGEBA	DDM	187	368	Y_800_ = 12.8	no rating	22.6	pHRR = 646 W/g THR = 23.9 kJ/g	[64]
TVEP	difuran diamine	162.7	275.4	Y_800_ = 30.0	V-0	28.5	THR-52.6%	[65]
PH-ODA-EP	DDM	204.9	280.1	Y_700_ = 41.7	V-0	40.5	pHRR-92.9% THR-72.0%	[66]
DGED	DDM	205	335	Y_800_ = 42.9	V-0	31.6	-	[67]
DGEL	DDS	314.4	379	Y_700_ = 44.0	V-0	32.5	pHRR-74.5% THR-16.5%	[68]
DGEG	DDM	223	-	Y_800_ = 43.8	V-0	33.1	pHRR-36.9% THR-48.5%	[69]
DGEEA	DDM	220	262	Y_800_ = 38.5	V-0	40.0	pHRR-84.9% THR-70.1%	[70]
HMF-GU-EP	difuran diamine	222	283	Y_800_ = 55.8	V-0	39.5	pHRR-92.4% THR-73.1%	[71]
GSPZ-EP	DDM	187	331	Y_700_ = 42.3	V-0	-	pHRR-60.5% THR-54.4%	[72]
THMT-EP	DDS	300	394	Y_800_ = 44.8	V-0	35.4	pHRR-65.5% THR-29.3%	[73]
DGEBA	DDS	180	391	Y_800_ = 13.5	no rating	22.9	pHRR = 423 W/g THR = 27.3 kJ/g	[73]
Triazole-VA-EP	DDM	135	-	Y_800_ = 34.7	V-0	39.5	pHRR-82.3% THR-52.8%	[74]

Note: Y_x_ indicates char yield at a certain temperature, and T_d5%_ indicates decomposition temperature at 5% weight loss; THR and pHRR values are shown by the reduced ratio compared to the DGEBA counterparts. The red part shows the research data of the DGEBA-based epoxy resin in the corresponding references.

**Table 2:** Comparative analysis between these bio-based resins and traditional petroleum-based epoxy resin.

Epoxy Resin	Curing Agent	*T*_g_	T_x%_ (°C)	E′ (30 °C, MPa)	Flame Retardancy	Antibacterial Rate		Ref.
						*S. aureus*	*E. coli*	
SA-GA-EP	DIFFA	204	T_10%_ = 300	2188.7	V-0	antibacterial	antibacterial	[155]
MGOL-EP-SC	-	265	*T*_d5%_ = 360 T_10%_ = 388	3789	low flammability	48.4%	-	[157]
MGOL-EP	DDM	204	*T*_d5%_ = 347 T_10%_ = 378	3884	low flammability	31.98%	-	[157]
DGEBA	DDM	189	*T*_d5%_ = 383 T_10%_ = 388	1727	no rating, flammable	13.1%		[157]

## Abbreviation

Abbreviations	Full names or Interpretation of abbreviations
DGEBA	Diglycidyl ether bisphenol A
BPA	Bisphenol A
ECH	Epichlorohydrin
TEIA	A trifunctional epoxy resin from itaconic acid
*T*_g_	Glass transition temperature
m-CPBA	m-chloroperbenzoic acid
DOPO	9,10-dihydro-9-oxo-10-phosphaphenanthrene-10-oxide
HCCP	Hexachlorocyclotriphosphazene
DGEBDB	A bio-based epoxy monomer containing DOPO units from vanillin and guaiacol
DDM	4,4-diaminodiphenyl methane
D230	Poly(propylene glycol) bis(2-aminopropyl ether) (D230, *M*_n_ 230 g/mol)
E51	commercial epoxy resin
PPDEG-EP	A novel epoxy monomer from eugenol and phenylphosphonic dichloride
*E*_a_	Activation energy
EP1 and EP2	Two epoxy monomers from vanillin, two diamines and diethyl phosphate.
DGEM	A fully bio-based epoxy resin precursor (DGEM) was synthesized from a naturally occurring magnolol through a highly efficient one step process
DDS	4, 4′-diaminodiphenyl sulfone
MTEP	A bio-based tetra-functional epoxy resin from a sustainable biomass feedstock, magnolol
DGEA	An apigenin-based epoxy monomer
LOI	Limiting oxygen index
OmbFdE	A bis-furan diepoxide
TEGA	2,2′-(ethane-1,2-diylbis(oxy)) bis(ethan-1-amine)
MI	A dicarboxylic acid oligomer from isosorbate and maleic anhydride
ESS	epoxidized sucrose soybean meal
TEP	A bio-based triepoxy from vanillin and guaiacol
FPAE	Rosin derivative
C-FPAE	A bio-based epoxy vitrimer containing dynamic reversible boronic ester bonds from FPAE
BDB	2,2′-(1,4-phenylene)-bis(4-mercaptan-1,3,2-dioxaborolane)
EHCPP	A cardanol-derived epoxy monomer
DMA	Dynamic mechanical analysis
VAN-AP	An imine-embedded bisphenol intermediate
GE-VAN-AP	An epoxy monomer from vanillin and aminophenol used VAN-AP
Get	Glycerol triglycidyl ether
CF	Carbon fiber
DADE	An epoxy monomer containing aldehyde group from vanillin
DADE-D230	a polymer network that cured DADE with D230
HMDO	A bisphenol of HMDO containing acetal from natural resources of vanillin and glycerol
D-A reaction	Diels-Alder reaction
FRP	Fiber reinforced plastic
MDS-EPO	The epoxy resin containing dynamic disulfide bond
MDA-EPO	The epoxy resin cured by traditional curing agent
ESO	Epoxidized soybean oil
ELO	Epoxidized linseed oil
ERSO	Epoxidized rubber seed oil
EOO	Epoxidized olive oi
SMPs	Shape memory polymers
Car-DCP-MAH	A cardanol-derived curing agent
Car-DPC-MAH	A cardanol-derived curing agent
PCM	Phase change material
EETS	An epoxy monomer (EETS) from eugenol and 1,1,3,3-tetramethyldisiloxane
APDS	4-aminophenyl disulfide
FPA	Fumarhippoic acid
MGOL-EP	A bio-based epoxy monomer from natural magnolol
MGOL-EP-SC	A fully bio-based and high-performance epoxy thermosetting resin
DIFFA	A furan-derived amine
SA-GA-EP/DIFFA	A fully bio-based epoxy thermosetting resin from syringaldehyde-derived Schiff base epoxy monomer that cured with DIFFA
GDE	The bio-based epoxy of glycerol diglycidyl ether
HVPA	A novel vanillin-derived curing agent containing Schiff base
HBE	Dihydrazone-containing epoxide monomers by condensation of vanillin and hydrazine hydrate

## References

[1] Liu J., Zhang L., Shun W., Dai J., Peng Y., Liu X. (2021). Recent development on bio-based thermosetting resins. J. Polym. Sci..

[2] Liu J., Peng Y., Zhao W., Jiang Y., Liu X. (2020). Research progress of bio-based thermal Resin. Thermosetting Resin.

[3] Kumar S., Samal S.K., Mohanty S., Nayak S.K. (2016). Recent development of biobased epoxy resins: A Review. Polym.-Plast. Technol..

[4] Kumar S., Krishnan S., Mohanty S., Nayak S.K. (2018). Synthesis and characterization of petroleum and biobased epoxy resins: A review. Polym. Int..

[5] Mustapha R., Rahmat A.R., Abdul Majid R., Mustapha S.N.H. (2019). Vegetable oil-based epoxy resins and their composites with bio-based hardener: A short review. Polym.-Plast. Technol. Mater..

[6] Fei X., Jia Y., Yang R., Lu G., Ma Y. (2020). Research progress of bio-based phenolic synthetic resins. Polym. Bull..

[7] Zhao X., Hou G., Yu S. (2022). Progress on research of plant phenolic bio-based epoxy resin. Polym. Mat. Sci. Eng..

[8] Santacesaria E., Tesser R., Di Serio M., Casale L., Verde D. (2010). New process for producing epichlorohydrin via glycerol chlorination. Ind. Eng. Chem. Res..

[9] Ma Y., Liu H., Wu J., Yuan L., Wang Y., Du X., Wang R., Marwa P.W., Petlulu P., Chen X. (2019). The adverse health effects of bisphenol A and related toxicity mechanisms. Environ. Res..

[10] Zhang C., Xue J., Yang X., Ke Y., Ou R., Wang Y., Madbouly S.A., Wang Q. (2022). From plant phenols to novel bio-based polymers. Prog. Polym. Sci..

[11] Liu J., Wang S., Peng Y., Zhu J., Zhao W., Liu X. (2021). Advances in sustainable thermosetting resins: From renewable feedstock to high performance and recyclability. Prog. Polym. Sci..

[12] Wan J., Zhao J., Zhang X., Fan H., Zhang J., Hu D., Jin P., Wang D.-Y. (2020). Epoxy thermosets and materials derived from bio-based monomeric phenols: Transformations and performances. Prog. Polym. Sci..

[13] Zhu Y., Romain C., Williams C.K. (2016). Sustainable polymers from renewable resources. Nature.

[14] Gonçalves F.A.M.M., Santos M., Cernadas T., Ferreira P., Alves P. (2021). Advances in the development of biobased epoxy resins: Insight into more sustainable materials and future applications. Int. Mater. Rev..

[15] Shibata M., Ohkita T. (2017). Fully biobased epoxy resin systems composed of a vanillin-derived epoxy resin and renewable phenolic hardeners. Eur. Polym. J..

[16] Zhang Y., Zhai M., Ma F., Li Y., Lyu B., Liu T., Gao Z., Wang L., Vincent D., Kessler M.R. (2021). Fully eugenol-based epoxy thermosets: Synthesis, curing, and properties. Macromol. Mater. Eng..

[17] Brocas A.-L., Llevot A., Mantzaridis C., Cendejas G., Auvergne R., Caillol S., Carlotti S., Cramail H. (2013). Epoxidized rosin acids as co-precursors for epoxy resins. Des. Monomers Polym..

[18] Ma S., Liu X., Fan L., Jiang Y., Cao L., Tang Z., Zhu J. (2014). Synthesis and properties of a bio-based epoxy resin with high epoxy value and low viscosity. ChemSusChem.

[19] Gonçalves F.A.M.M., Ferreira P., Alves P. (2021). Synthesis and characterization of itaconic-based epoxy resin: Chemical and thermal properties of partially biobased epoxy resins. Polymer.

[20] Janvier M., Hollande L., Jaufurally A.S., Pernes M., Menard R., Grimaldi M., Beaugrand J., Balaguer P., Ducrot P.H., Allais F. (2017). Syringaresinol: A renewable and safer alternative to bisphenol A for epoxy-amine resins. ChemSusChem.

[21] Wei J., Duan Y., Wang H., Hui J., Qi J. (2022). Bio-based trifunctional diphenolic acid epoxy resin with high *T*_g_ and low expansion coefficient: Synthesis and properties. Polym. Bull..

[22] Savonnet E., Grau E., Grelier S., Defoort B., Cramail H. (2018). Divanillin-based epoxy precursors as DGEBA substitutes for biobased epoxy thermosets. ACS Sustain. Chem. Eng..

[23] Ye J., Ma S., Wang B., Chen Q., Huang K., Xu X., Li Q., Wang S., Lu N., Zhu J. (2021). High-performance bio-based epoxies from ferulic acid and furfuryl alcohol: Synthesis and properties. Green Chem..

[24] Garrison M.D., Savolainen M.A., Chafin A.P., Baca J.E., Bons A.M., Harvey B.G. (2020). Synthesis and characterization of high-performance, bio-based epoxy-amine networks derived from resveratrol. ACS Sustain. Chem. Eng..

[25] Bu M., Zhang X., Zhou T., Lei C. (2022). Fully bio-based epoxy resins derived from magnolol and varying furan amines: Cure kinetics, superior mechanical and thermal properties. Eur. Polym. J..

[26] Mora A.S., Decostanzi M., David G., Caillol S. (2019). Cardanol-based epoxy monomers for high thermal properties thermosets. Eur. J. Lipid Sci. Tech..

[27] Xu J., Liu X., Fu S. (2022). Bio-based epoxy resin from gallic acid and its thermosets toughened with renewable tannic acid derivatives. J. Mater. Sci..

[28] Meng J., Zeng Y., Chen P., Zhang J., Yao C., Fang Z., Guo K. (2019). New ultrastiff bio-furan epoxy networks with high *T*_g_: Facile synthesis to excellent properties. Eur. Polym. J..

[29] Wang X., Guo W., Song L., Hu Y. (2019). Intrinsically flame retardant bio-based epoxy thermosets: A review. Compos. Part B-Eng..

[30] Huo S., Song P., Yu B., Ran S., Chevali V.S., Liu L., Fang Z., Wang H. (2021). Phosphorus-containing flame retardant epoxy thermosets: Recent advances and future perspectives. Prog. Polym. Sci..

[31] Zhi M., Yang X., Fan R., Yue S., Zheng L., Liu Q., He Y. (2022). A comprehensive review of reactive flame-retardant epoxy resin: Fundamentals, recent developments, and perspectives. Polym. Degrad. Stab..

[32] Niu H., Wang X., Song L., Hu Y. (2022). Progress on intrinsically flame-retardant bio-based epoxy thermosets. Acta Polym. Sinica..

[33] Rashid M.A., Liu W., Wei Y., Jiang Q. (2022). Review of intrinsically recyclable biobased epoxy thermosets enabled by dynamic chemical bonds. Polym.-Plast. Tech. Mat..

[34] Liu Y., Yu Z., Wang B., Li P., Zhu J., Ma S. (2022). Closed-loop chemical recycling of thermosetting polymers and their applications: A review. Green Chem..

[35] Li Y., Wu Y., Li K., Lin H., Wang M., Zheng L., Wu C., Zhang X. (2024). Recycling of epoxy resins with degradable structures or dynamic cross-linking networks: A review. Ind. Eng. Chem. Res..

[36] Zhao W., Liu J., Wang S., Dai J., Liu X. (2024). Bio-based thermosetting resins: From molecular engineering to intrinsically multifunctional customization. Adv. Mater..

[37] Jin F.-L., Li X., Park S.-J. (2015). Synthesis and application of epoxy resins: A review. J. Ind. Eng. Chem..

[38] Xie R., Yuan Y., Sun P., Liu Z., Ma J., Yang G., Wang K., Li M., Shang L., Ao Y. (2022). Design of epoxy resin with sustainability, high adhesion and excellent flame retardancy based on bio-based molecules. J. Mater. Sci..

[39] Liu J., Dai J., Wang S., Peng Y., Cao L., Liu X. (2020). Facile synthesis of bio-based reactive flame retardant from vanillin and guaiacol for epoxy resin. Compos. Part B-Eng..

[40] Zhou J., Heng Z., Zhang H., Chen Y., Zou H., Liang M. (2019). High residue bio-based structural-functional integration epoxy and intrinsic flame retardant mechanism study. RSC Adv..

[41] Chi Z., Guo Z., Xu Z., Zhang M., Li M., Shang L., Ao Y. (2020). A DOPO-based phosphorus-nitrogen flame retardant bio-based epoxy resin from diphenolic acid: Synthesis, flame-retardant behavior and mechanism. Polym. Degrad. Stab..

[42] Yang W., Ding H., Liu T., Ou R., Lin J., Puglia D., Xu P., Wang Q., Dong W., Du M. (2021). Design of intrinsically flame-retardant vanillin-based epoxy resin for thermal-conductive epoxy/graphene aerogel composites. ACS Appl. Mater. Interfaces.

[43] Yang W., Ding H., Zhou W., Liu T., Xu P., Puglia D., Kenny J.M., Ma P. (2022). Design of inherent fire retarding and degradable bio-based epoxy vitrimer with excellent self-healing and mechanical reprocessability. Compos. Sci. Technol..

[44] Liu J., He Z., Wu G., Zhang X., Zhao C., Lei C. (2020). Synthesis of a novel nonflammable eugenol-based phosphazene epoxy resin with unique burned intumescent char. Chem. Eng. J..

[45] Huang J., Guo W., Wang X., Song L., Hu Y. (2021). Intrinsically flame retardant cardanol-based epoxy monomer for high-performance thermosets. Polym. Degrad. Stab..

[46] Zhou W., Lv D., Ding H., Xu P., Zhang C., Ren Y., Yang W., Ma P. (2022). Synthesis of eugenol-based phosphorus-containing epoxy for enhancing the flame-retardancy and mechanical performance of DGEBA epoxy resin. React. Funct. Polym..

[47] Ma C., Qian L., Li J. (2021). Effect of functional groups of magnolol-based cyclic phosphonate on structure and properties of flame retardant epoxy resin. Polym. Degrad. Stab..

[48] Zhong J., Huang Y., Chen Y., Li L., Guo C. (2022). Synthesis of eugenol-modified epoxy resin and application on wood flame retardant coating. Ind. Crop. Prod..

[49] Chen J., Zhang Y., He F., Ying J., Li S.N., Peng L., Wu Q., Fan Z., Jiang B. (2023). Facile synthesis of intrinsically flame-retardant epoxy thermosets with high mechanical properties from lignin derivatives. J. Appl. Polym. Sci..

[50] Xie W., Tang D., Liu S., Zhao J. (2020). Facile synthesis of bio-based phosphorus-containing epoxy resins with excellent flame resistance. Polym. Test..

[51] Miao J.T., Yuan L., Guan Q., Liang G., Gu A. (2018). Biobased epoxy resin derived from eugenol with excellent integrated performance and high renewable carbon content. Polym. Int..

[52] Faye I., Decostanzi M., Ecochard Y., Caillol S. (2017). Eugenol bio-based epoxy thermosets: From cloves to applied materials. Green Chem..

[53] Park H.-W., Toan M., Kim H.-J., Lee J.-H., Shin S. (2020). Renewable epoxy thermosets with extremely high biomass content from furan derivatives and their flame retardancy. J. Ind. Eng. Chem..

[54] Wang S., Ma S., Xu C., Liu Y., Dai J., Wang Z., Liu X., Chen J., Shen X., Wei J. (2017). Vanillin-derived high-performance flame retardant epoxy resins: Facile synthesis and properties. Macromolecules.

[55] Kumar B., Roy S., Agumba D.O., Pham D.H., Kim J. (2022). Effect of bio-based derived epoxy resin on interfacial adhesion of cellulose film and applicability towards natural jute fiber-reinforced composites. Int. J. Biol. Macromol..

[56] Liu X., Li M., Huang Y., Hu M., Li L. (2023). Facile preparation of magnolol-based epoxy resin with intrinsic flame retardancy, high rigidity and hydrophobicity. Ind. Crop. Prod..

[57] Wang X., Niu H., Guo W., Song L., Hu Y. (2021). Cardanol as a versatile platform for fabrication of bio-based flame-retardant epoxy thermosets as DGEBA substitutes. Chem. Eng. J..

[58] Qi Y., Weng Z., Zhang K., Wang J., Zhang S., Liu C., Jian X. (2020). Magnolol-based bio-epoxy resin with acceptable glass transition temperature, processability and flame retardancy. Chem. Eng. J..

[59] Qi Y., Weng Z., Kou Y., Li J., Cao Q., Wang J., Zhang S., Jian X. (2021). Facile synthesis of bio-based tetra-functional epoxy resin and its potential application as high-performance composite resin matrix. Compos. Part B-Eng..

[60] Lu C., Bian S., Hu K., Li C., Zheng K., Sun Q. (2022). Biomass-based epoxy resin derived from resveratrol with high temperature resistance and intrinsic flame retardant properties. Ind. Crop. Prod..

[61] Song X., Deng Z.-P., You C.-W., Sun R.-Y., Song F., Wang X.-L., Chen L., Wang Y.-Z. (2022). High-performance and fire-resistant epoxy thermosets derived from plant-derived ferulic acid. Ind. Crop. Prod..

[62] Song X., Deng Z.-P., Li C.-B., Song F., Wang X.-L., Chen L., Guo D.-M., Wang Y.-Z. (2022). A bio-based epoxy resin derived from p-hydroxycinnamic acid with high mechanical properties and flame retardancy. Chin. Chem. Lett..

[63] Zhang Y., Zhang X., Wang P., Liu Y., Wan M., Zhang K. (2023). Phosphorus-free curcumin-derived epoxy resin with exceptional flame retardancy, glass transition temperature and mechanical properties. Polym. Degrad. Stab..

[64] Li J., Weng Z., Cao Q., Qi Y., Lu B., Zhang S., Wang J., Jian X. (2022). Synthesis of an aromatic amine derived from biomass and its use as a feedstock for versatile epoxy thermoset. Chem. Eng. J..

[65] Niu H.-X., Yang T.-M., Wang X., Zhang P., Guo W., Song L., Hu Y. (2024). High biomass content, anti-flammable and degradable epoxy thermosets by curing a tyramine-derived epoxy monomer with a furan-derived diamine for non-destructively recyclable carbon fiber composite application. Green Chem..

[66] Xie W., Huang S., Tang D., Liu S., Zhao J. (2020). Biomass-derived Schiff base compound enabled fire-safe epoxy thermoset with excellent mechanical properties and high glass transition temperature. Chem. Eng. J..

[67] Dai J., Peng Y., Teng N., Liu Y., Liu C., Shen X., Mahmud S., Zhu J., Liu X. (2018). High-performing and fire-resistant biobased epoxy resin from renewable sources. ACS Sustain. Chem. Eng..

[68] Gao T.-Y., Wang F.-D., Xu Y., Wei C.-X., Zhu S.-E., Yang W., Lu H.-D. (2022). Luteolin-based epoxy resin with exceptional heat resistance, mechanical and flame retardant properties. Chem. Eng. J..

[69] Dai J., Teng N., Liu J., Feng J., Zhu J., Liu X. (2019). Synthesis of bio-based fire-resistant epoxy without addition of flame retardant elements. Compos. Part B-Eng..

[70] Nabipour H., Qiu S., Wang X., Song L., Hu Y. (2021). Phosphorus-free ellagic acid-derived epoxy thermosets with intrinsic antiflammability and high glass transition temperature. ACS Sustain. Chem. Eng..

[71] Nabipour H., Wang X., Song L., Hu Y. (2023). Synthesis of a bio-based and intrinsically anti-flammable epoxy thermoset and the application of its carbonized foam as an efficient CO_2_ capture adsorbent. Mater. Today Sustain..

[72] Qi Y., Wang J., Kou Y., Pang H., Zhang S., Li N., Liu C., Weng Z., Jian X. (2019). Synthesis of an aromatic N-heterocycle derived from biomass and its use as a polymer feedstock. Nat. Commun..

[73] Qi Y., Weng Z., Kou Y., Song L., Li J., Wang J., Zhang S., Liu C., Jian X. (2021). Synthesize and introduce bio-based aromatic s-triazine in epoxy resin: Enabling extremely high thermal stability, mechanical properties, and flame retardancy to achieve high-performance sustainable polymers. Chem. Eng. J..

[74] Niu H., Nabipour H., Wang X., Song L., Hu Y. (2021). Phosphorus-free vanillin-derived intrinsically flame-retardant epoxy thermoset with extremely low heat release rate and smoke emission. ACS Sustain. Chem. Eng..

[75] Liu S.H., Zhang X.Q., Liu J.H., Lei C.H., Dong Z.X. (2021). A novel bio-based epoxy resin from oligomer: Excellent processability, high heat resistance, and intrinsic flame retardancy. Express Polym. Lett..

[76] Liu S., Zhang X., Bu M., Lei C. (2022). Properties tailoring of biobased epoxy resins by regulating the degree of polymerization of oligomers. Eur. Polym. J..

[77] Meng J., Chen P., Yang R., Dai L., Yao C., Fang Z., Guo K. (2021). Thermal stable honokiol-derived epoxy resin with reinforced thermal conductivity, dielectric properties and flame resistance. Chem. Eng. J..

[78] Tian Y., Wang Q., Shen L., Cui Z., Kou L., Cheng J., Zhang J. (2020). A renewable resveratrol-based epoxy resin with high *T*_g_, excellent mechanical properties and low flammability. Chem. Eng. J..

[79] Tian Y., Ke M., Wang X., Wu G., Zhang J., Cheng J. (2021). A resveratrol-based epoxy resin with ultrahigh *T*_g_ and good processability. Eur. Polym. J..

[80] Wang Z., Zhang X., Cai J., Xie J. (2023). Plant-derived *p*-hydroxyphenylacrylic acid-derived epoxy resins exhibit excellent flame retardancy, hydrophobicity, degradability, and low dielectric loss after curing with bio-based fluorinated Schiff bases. Polym. Degrad. Stab..

[81] Wan J., Gan B., Li C., Molina-Aldareguia J., Kalali E.N., Wang X., Wang D.-Y. (2016). A sustainable, eugenol-derived epoxy resin with high biobased content, modulus, hardness and low flammability: Synthesis, curing kinetics and structure–property relationship. Chem. Eng. J..

[82] Wan J., Gan B., Li C., Molina-Aldareguia J., Li Z., Wang X., Wang D.-Y. (2015). A novel biobased epoxy resin with high mechanical stiffness and low flammability: Synthesis, characterization and properties. J. Mater. Chem. A.

[83] Zhang D., Jin S., Wan J., Wang J., Li Y., Jin P., Hu D. (2021). A dieugenol-based epoxy monomer with high bio-based content, low viscosity and low flammability. Mater. Today Commun..

[84] Wang X., Nabipour H., Kan Y.-C., Song L., Hu Y. (2022). A fully bio-based, anti-flammable and non-toxic epoxy thermosetting network for flame-retardant coating applications. Prog. Org. Coat..

[85] Nabipour H., Wang X., Song L., Hu Y. (2022). Intrinsically anti-flammable apigenin-derived epoxy thermosets with high glass transition temperature and mechanical strength. Chin. J. Polym. Sci..

[86] Du Y., Zhao G., Shi G., Wang Y., Li W., Ren S. (2022). Effect of crosslink structure on mechanical properties, thermal stability and flame retardancy of natural flavonoid based epoxy resins. Eur. Polym. J..

[87] Meng J., Zeng Y., Zhu G., Zhang J., Chen P., Cheng Y., Fang Z., Guo K. (2019). Sustainable bio-based furan epoxy resin with flame retardancy. Polym. Chem..

[88] Miao J.-T., Yuan L., Guan Q., Liang G., Gu A. (2017). Biobased heat resistant epoxy resin with extremely high biomass content from 2,5-furandicarboxylic acid and eugenol. ACS Sustain. Chem. Eng..

[89] Meng J., Zeng Y., Chen P., Zhang J., Yao C., Fang Z., Ouyang P., Guo K. (2019). Flame retardancy and mechanical properties of bio-based furan epoxy resins with high crosslink density. Macromol. Mater. Eng..

[90] Nabipour H., Wang X., Song L., Hu Y. (2023). A furan-derived epoxy thermoset with inherent anti-flammability, degradability, and raw material recycling. Mater. Today Chem..

[91] Nabipour H., Wang X., Kandola B., Song L., Kan Y., Chen J., Hu Y. (2023). A bio-based intrinsically flame-retardant epoxy vitrimer from furan derivatives and its application in recyclable carbon fiber composites. Polym. Degrad. Stab..

[92] Ma S., Webster D.C. (2015). Naturally occurring acids as cross-linkers to yield VOC-free, high-performance, fully bio-based, degradable thermosets. Macromolecules.

[93] Liu T., Guo X., Liu W., Hao C., Wang L., Hiscox W.C., Liu C., Jin C., Xin J., Zhang J. (2017). Selective cleavage of ester linkages of anhydride-cured epoxy using a benign method and reuse of the decomposed polymer in new epoxy preparation. Green Chem..

[94] Zhong L., Hao Y., Zhang J., Wei F., Li T., Miao M., Zhang D. (2022). Closed-loop recyclable fully bio-based epoxy vitrimers from ferulic acid-derived hyperbranched epoxy resin. Macromolecules.

[95] Zhou S., Huang K., Xu X., Wang B., Zhang W., Su Y., Hu K., Zhang C., Zhu J., Weng G. (2023). Rigid-and-flexible, degradable, fully biobased thermosets from lignin and soybean oil: Synthesis and properties. ACS Sustain. Chem. Eng..

[96] Ma S., Webster D.C., Jabeen F. (2016). Hard and Flexible, Degradable thermosets from renewable bioresources with the assistance of water and ethanol. Macromolecules.

[97] Yan X., Liu T., Hao C., Shao L., Chang Y.-C., Cai Z., Shang S., Song Z., Zhang J. (2023). Rosin derived catalyst-free vitrimer with hydrothermal recyclability and application in high performance fiber composite. Ind. Crop. Prod..

[98] Di Mauro C., Genua A., Mija A. (2022). Fully bio-based reprocessable thermosetting resins based on epoxidized vegetable oils cured with itaconic acid. Ind. Crop. Prod..

[99] Liu T., Hao C., Wang L., Li Y., Liu W., Xin J., Zhang J. (2017). Eugenol-derived biobased epoxy: Shape memory, repairing, and recyclability. Macromolecules.

[100] Liu T., Hao C., Zhang S., Yang X., Wang L., Han J., Li Y., Xin J., Zhang J. (2018). A self-healable high glass transition temperature bioepoxy material based on vitrimer chemistry. Macromolecules.

[101] Feng X., Fan J., Li A., Li G. (2019). Biobased tannic acid cross-linked epoxy thermosets with hierarchical molecular structure and tunable properties: Damping, shape memory, and recyclability. ACS Sustain. Chem. Eng..

[102] Cao L., Fan J., Huang J., Chen Y. (2019). A robust and stretchable cross-linked rubber network with recyclable and self-healable capabilities based on dynamic covalent bonds. J. Mater. Chem. A.

[103] Kadam A., Pawar M., Yemul O., Thamke V., Kodam K. (2015). Biodegradable biobased epoxy resin from karanja oil. Polymer.

[104] Liu T., Zhang S., Hao C., Verdi C., Liu W., Liu H., Zhang J. (2019). Glycerol induced catalyst-free curing of epoxy and vitrimer preparation. Macromol. Rapid Commun..

[105] Xu Y.-z., Fu P., Dai S.-l., Zhang H.-b., Bi L.-w., Jiang J.-x., Chen Y.-x. (2021). Catalyst-free self-healing fully bio-based vitrimers derived from tung oil: Strong mechanical properties, shape memory, and recyclability. Ind. Crop. Prod..

[106] Wu J., Yu X., Zhang H., Guo J., Hu J., Li M.-H. (2020). Fully biobased vitrimers from glycyrrhizic acid and soybean oil for self-healing, shape memory, weldable, and recyclable materials. ACS Sustain. Chem. Eng..

[107] Xu Y., Dai S., Bi L., Jiang J., Zhang H., Chen Y. (2022). Catalyst-free self-healing bio-based vitrimer for a recyclable, reprocessable, and self-adhered carbon fiber reinforced composite. Chem. Eng. J..

[108] Li Y., Liu T., Zhang S., Shao L., Fei M., Yu H., Zhang J. (2020). Catalyst-free vitrimer elastomers based on a dimer acid: Robust mechanical performance, adaptability and hydrothermal recyclability. Green Chem..

[109] Xu Y., Dai S., Bi L., Jiang J., Zhang H., Chen Y. (2021). Catalyst-free self-healing bio-based polymers: Robust mechanical properties, shape memory, and recyclability. J. Agric. Food Chem..

[110] Zeng Y., Li J., Liu S., Yang B. (2021). Rosin-based epoxy vitrimers with dynamic boronic ester bonds. Polymers.

[111] Chen Y., Tang Z., Liu Y., Wu S., Guo B. (2019). Mechanically robust, self-healable, and reprocessable elastomers enabled by dynamic dual cross-links. Macromolecules.

[112] Ke Y., Yang X., Chen Q., Xue J., Song Z., Zhang Y., Madbouly S.A., Luo Y., Li M., Wang Q. (2021). Recyclable and fluorescent epoxy polymer networks from cardanol via solvent-free epoxy-thiol chemistry. ACS Appl. Polym. Mater..

[113] Ma J., Li G., Hua X., Liu N., Liu Z., Zhang F., Yu L., Chen X., Shang L., Ao Y. (2022). Biodegradable epoxy resin from vanillin with excellent flame-retardant and outstanding mechanical properties. Polym. Degrad. Stab..

[114] Guo Z., Liu B., Zhou L., Wang L., Majeed K., Zhang B., Zhou F., Zhang Q. (2020). Preparation of environmentally friendly bio-based vitrimers from vanillin derivatives by introducing two types of dynamic covalent C=N and S-S bonds. Polymer.

[115] Song F., Li Z., Jia P., Zhang M., Bo C., Feng G., Hu L., Zhou Y. (2019). Tunable “soft and stiff”, self-healing, recyclable, thermadapt shape memory biomass polymers based on multiple hydrogen bonds and dynamic imine bonds. J. Mater. Chem. A.

[116] Zhao S., Abu-Omar M.M. (2018). Recyclable and malleable epoxy thermoset bearing aromatic imine bonds. Macromolecules.

[117] Liu X., Liang L., Lu M., Song X., Liu H., Chen G. (2020). Water-resistant bio-based vitrimers based on dynamic imine bonds: Self-healability, remodelability and ecofriendly recyclability. Polymer.

[118] Yu Q., Peng X., Wang Y., Geng H., Xu A., Zhang X., Xu W., Ye D. (2019). Vanillin-based degradable epoxy vitrimers: Reprocessability and mechanical properties study. Eur. Polym. J..

[119] Liu Y.-Y., Liu G.-L., Li Y.-D., Weng Y., Zeng J.-B. (2021). Biobased high-performance epoxy vitrimer with UV shielding for recyclable carbon fiber reinforced composites. ACS Sustain. Chem. Eng..

[120] Memon H., Liu H., Rashid M.A., Chen L., Jiang Q., Zhang L., Wei Y., Liu W., Qiu Y. (2020). Vanillin-based epoxy vitrimer with high performance and closed-loop recyclability. Macromolecules.

[121] Su X., Zhou Z., Liu J., Luo J., Liu R. (2020). A recyclable vanillin-based epoxy resin with high-performance that can compete with DGEBA. Eur. Polym. J..

[122] Wang S., Ma S., Li Q., Yuan W., Wang B., Zhu J. (2018). Robust, Fire-Safe, Monomer-recovery, highly malleable thermosets from renewable bioresources. Macromolecules.

[123] Xie W., Huang S., Liu S., Zhao J. (2021). Imine-functionalized biomass-derived dynamic covalent thermosets enabled by heat-induced self-crosslinking and reversible structures. Chem. Eng. J..

[124] Xu X., Ma S., Wu J., Yang J., Wang B., Wang S., Li Q., Feng J., You S., Zhu J. (2019). High-performance, command-degradable, antibacterial Schiff base epoxy thermosets: Synthesis and properties. J. Mater. Chem. A.

[125] Yuan W., Ma S., Wang S., Li Q., Wang B., Xu X., Huang K., Chen J., You S., Zhu J. (2019). Synthesis of fully bio-based diepoxy monomer with dicyclo diacetal for high-performance, readily degradable thermosets. Eur. Polym. J..

[126] Ma S., Wei J., Jia Z., Yu T., Yuan W., Li Q., Wang S., You S., Liu R., Zhu J. (2019). Readily recyclable, high-performance thermosetting materials based on a lignin-derived spiro diacetal trigger. J. Mater. Chem. A.

[127] Wang B., Ma S., Li Q., Zhang H., Liu J., Wang R., Chen Z., Xu X., Wang S., Lu N. (2020). Facile synthesis of “digestible”, rigid-and-flexible, bio-based building block for high-performance degradable thermosetting plastics. Green Chem..

[128] Karami Z., Zohuriaan-Mehr M.J., Rostami A. (2017). Bio-based thermo-healable non-isocyanate polyurethane DA network in comparison with its epoxy counterpart. J. CO2 Util..

[129] Shen X., Liu X., Wang J., Dai J., Zhu J. (2017). Synthesis of an epoxy monomer from bio-based 2,5-furandimethanol and its toughening via Diels-Alder reaction. Ind. Eng. Chem. Res..

[130] Lejeail M., Fischer H.R. (2020). Investigations on the replacement of bismaleimide by the bio-based bisitaconimide for recyclable thermoset composites based on thermo-reversible Diels-Alder cross-links. Eur. Polym. J..

[131] Wu P., Liu L., Wu Z. (2020). Synthesis of Diels-Alder reaction-based remendable epoxy matrix and corresponding self-healing efficiency to fibrous composites. Macromol. Mater. Eng..

[132] Zhang Y., Ye J., Qu D., Wang H., Chai C., Feng L. (2021). Thermo-adjusted self-healing epoxy resins based on Diels–Alder dynamic chemical reaction. Polym. Eng. Sci..

[133] Zhao H., Feng L., Shi X., Wang Y., Liu Y. (2018). Synthesis and healing behavior of thermo-reversible self-healing epoxy resins. Acta Polym. Sin..

[134] Liu Z., Zhu X., Tian Y., Zhou K., Cheng J., Zhang J. (2022). Bio-based recyclable Form-Stable phase change material based on thermally reversible Diels–Alder reaction for sustainable thermal energy storage. Chem. Eng. J..

[135] Mauro C.D., Genua A., Mija A. (2020). Building thermally and chemically reversible covalent bonds in vegetable oil based epoxy thermosets. Influence of epoxy–hardener ratio in promoting recyclability. Mater. Adv..

[136] Ocando C., Ecochard Y., Decostanzi M., Caillol S., Avérous L. (2020). Dynamic network based on eugenol-derived epoxy as promising sustainable thermoset materials. Eur. Polym. J..

[137] Shan S., Mai D., Lin Y., Zhang A. (2021). Self-Healing, Reprocessable, and Degradable bio-based epoxy elastomer bearing aromatic disulfide bonds and its application in strain sensors. ACS Appl. Polym. Mater..

[138] Ma Z., Wang Y., Zhu J., Yu J., Hu Z. (2017). Bio-based epoxy vitrimers: Reprocessibility, controllable shape memory, and degradability. J. Polym. Sci. Part A Polym. Chem..

[139] Zhang C., Wang X., Liang D., Deng H., Lin Z., Feng P., Wang Q. (2021). Rapid self-healing, multiple recyclability and mechanically robust plant oil-based epoxy resins enabled by incorporating tri-dynamic covalent bonding. J. Mater. Chem. A.

[140] Zeng Y., Yang B., Yuan R., Luo Z. (2023). Fabrication and properties of rosin-based epoxy vitrimer with dual dynamic covalent bonds. Chem. J. Chin. Univ..

[141] di Mauro C., Tran T.-N., Graillot A., Mija A. (2020). Enhancing the recyclability of a vegetable oil-based epoxy thermoset through initiator influence. ACS Sustain. Chem. Eng..

[142] Zheng N., Xu Y., Zhao Q., Xie T. (2021). Dynamic covalent polymer networks: A molecular platform for designing functions beyond chemical recycling and self-healing. Chem. Rev..

[143] Herath M., Epaarachchi J., Islam M., Fang L., Leng J. (2020). Light activated shape memory polymers and composites: A review. Eur. Polym. J..

[144] Rodriguez J.N., Zhu C., Duoss E.B., Wilson T.S., Spadaccini C.M., Lewicki J.P. (2016). Shape-morphing composites with designed micro-architectures. Sci. Rep..

[145] Taung Mai L.L., Aung M.M., Muhamad Saidi S.A., H’Ng P.S., Rayung M., Jaafar A.M. (2021). Non edible oil-based epoxy resins from jatropha oil and their shape memory behaviors. Polymers.

[146] Huang J.-L., Ding H.-L., Wang X., Song L., Hu Y. (2022). Cardanol-derived anhydride cross-linked epoxy thermosets with intrinsic anti-flammability, toughness and shape memory effect. Chem. Eng. J..

[147] Lu C., Wang X., Shen Y., Wang J., Yong Q., Chu F. (2022). Fabrication of sustainable, toughening epoxy thermosets with rapidly thermal and light-triggered shape memory property. J. Polym. Sci..

[148] Amornkitbamrung L., Srisaard S., Jubsilp C., Bielawski C.W., Um S.H., Rimdusit S. (2020). Near-infrared light responsive shape memory polymers from bio-based benzoxazine/epoxy copolymers produced without using photothermal filler. Polymer.

[149] Lu C., Liu Y., Wang C., Yong Q., Wang J., Chu F. (2022). An integrated strategy to fabricate bio-based dual-cure and toughened epoxy thermosets with photothermal conversion property. Chem. Eng. J..

[150] Li W., Xiao L., Wang Y., Chen J., Nie X. (2021). Self-healing silicon-containing eugenol-based epoxy resin based on disulfide bond exchange: Synthesis and structure-property relationships. Polymer.

[151] Yang X., Guo L., Xu X., Shang S., Liu H. (2020). A fully bio-based epoxy vitrimer: Self-healing, triple-shape memory and reprocessing triggered by dynamic covalent bond exchange. Mater. Des..

[152] Oh Y., Lee K.M., Jung D., Chae J.A., Kim H.J., Chang M., Park J.J., Kim H. (2019). Sustainable, naringenin-based thermosets show reversible macroscopic shape changes and enable modular recycling. ACS Macro. Lett..

[153] Di Mauro C., Malburet S., Graillot A., Mija A. (2020). Recyclable, repairable, and reshapable (3R) thermoset materials with shape memory properties from bio-based epoxidized vegetable oils. Appl. Bio. Mater..

[154] Podgorski M., Fairbanks B.D., Kirkpatrick B.E., McBride M., Martinez A., Dobson A., Bongiardina N.J., Bowman C.N. (2020). Toward stimuli-responsive dynamic thermosets through continuous development and improvements in covalent adaptable networks (CANs). Adv. Mater..

[155] Feng H., Hu J., Jin D., Wang S., Dai J., Liu X. (2022). Bio-based Epoxy Resin: Controllable Degradation, Chemical Recovery and Antimicrobial Property. Acta Polym. Sin..

[156] Nabipour H., Wang X., Song L., Hu Y. (2021). A high performance fully bio-based epoxy thermoset from a syringaldehyde-derived epoxy monomer cured by furan-derived amine. Green Chem..

[157] Cao Q., Weng Z., Qi Y., Li J., Liu W., Liu C., Zhang S., Wei Z., Chen Y., Jian X. (2022). Achieving higher performances without an external curing agent in natural magnolol-based epoxy resin. Chin. Chem. Lett..

[158] Huang K., Fan X., Ashby R., Ngo H. (2021). Structure-activity relationship of antibacterial bio-based epoxy polymers made from phenolic branched fatty acids. Prog. Org. Coat..

[159] Modjinou T., Versace D.-L., Abbad-Andaloussi S., Langlois V., Renard E. (2017). Antibacterial and antioxidant photoinitiated epoxy co-networks of resorcinol and eugenol derivatives. Mater. Today Commun..

[160] Chen M.-X., Dai J.-Y., Zhang L.-Y., Wang S.-P., Liu J.-K., Wu Y.-G., Ba X.-W., Liu X.-Q. (2023). The role of renewable protocatechol acid in epoxy coating modification: Significantly improved antibacterial and adhesive properties. Chin. J. Polym. Sci..

[161] Lobiuc A., Paval N.-E., Mangalagiu I.I., Gheorghitã R., Teliban G.-C., Amariucai-Mantu D., Stoleru V. (2023). Improving the antimicrobial and mechanical properties of epoxy resins via nanomodification: An Overview. Molecules.

[162] Ji J., Huang S., Liu S., Yuan Y., Zhao J., Zhang S. (2022). A novel biomass-derived Schiff base waterborne epoxy coating for flame retardation and anti-bacteria. Polym. Degrad. Stab..

[163] Liu Y., Yu Z., Lu G., Chen W., Ye Z., He Y., Tang Z., Zhu J. (2023). Versatile levulinic acid-derived dynamic covalent thermosets enabled by in situ generated imine and multiple hydrogen bonds. Chem. Eng. J..

[164] Peng J., Xie S., Liu T., Wang D., Ou R., Guo C., Wang Q., Liu Z. (2022). High-performance epoxy vitrimer with superior self-healing, shape-memory, flame retardancy, and antibacterial properties based on multifunctional curing agent. Compos. Part B-Eng..

[165] Xu X., Ma S., Wang S., Wu J., Li Q., Lu N., Liu Y., Yang J., Feng J., Zhu J. (2020). Dihydrazone-based dynamic covalent epoxy networks with high creep resistance, controlled degradability, and intrinsic antibacterial properties from bioresources. J. Mater. Chem. A.

[166] Ceramella J., Iacopetta D., Catalano A., Cirillo F., Lappano R., Sinicropi M.S. (2022). A review on the antimicrobial activity of Schiff bases: Data collection and recent studies. Antibiotics.

